# Dynamic Semantic World Models and Increased Situational Awareness for Highly Automated Inland Waterway Transport

**DOI:** 10.3389/frobt.2021.739062

**Published:** 2022-01-17

**Authors:** Senne Van Baelen, Gerben Peeters, Herman Bruyninckx, Paolo Pilozzi, Peter Slaets

**Affiliations:** ^1^ Department of Mechanical Engineering, KU Leuven, Leuven, Belgium; ^2^ Faculty of Mechanical Engineering, TU Eindhoven, Eindhoven, Netherlands; ^3^ Flanders Make – Leuven, Leuven, Belgium

**Keywords:** inland navigation, modelling, situational awareness, semantic map, world model, navigation charts, COLREG

## Abstract

Automated surface vessels must integrate many tasks and motions at the same time. Moreover, vessels as well as monitoring and control services need to react to physical disturbances, to dynamically allocate software resources available within a particular environment, and to communicate with various other actors in particular navigation and traffic situations. In this work, the responsibility for the situational awareness is given to a mediator that decides *how*: 1) to assess the impact of the actual physical environment on the quality and performance of the ongoing task executions; 2) to make sure these tasks satisfy the system requirements; and 3) to be robust against disturbances. This paper proposes a set of semantic world models within the context of inland waterway transport, and discusses policies and methodologies to compose, use, and connect these models. Model-conform entities and relations are composed dynamically, that is, corresponding to the opportunities and challenges offered by the actual situation. The semantic world models discussed in this work are divided into two main categories: 1) the semantic description of a vessel’s *own* properties and relationships, called the *internal world model*, or body model, and 2) the semantic description of its local environment, called the *external world model*, or map. A range of experiments illustrate the potential of using such models to decide the reactions of the application at runtime. Furthermore, three dynamic, context-dependent, ship domains are integrated in the map as two-dimensional geometric entities around a moving vessel to increase the situational awareness of automated vessels. Their geometric representations depend on the associated relations; for example, with: 1) the motion of the vessel, 2) the actual, desired, or hypothesised tasks, 3) perception sensor information, and 4) other geometries, e.g., features from the Inland Electronic Navigational Charts. The ability to unambiguously understand the environmental context, as well as the motion or position of surrounding entities, allows for resource-efficient and straightforward control decisions. The semantic world models facilitate knowledge sharing between actors, and significantly enhance explainability of the actors’ behaviour and control decisions.

## 1 Introduction

The European Inland Waterway Transport (IWT) sector has a substantial yet under-exploited potential. A dense and distributed network of rivers and canals flows through the European hinterland, especially in the more northern areas. Compared to the dominating European road-based freight transport, the IWT sector has lower external costs (European Commission, 2019; van Essen, 2018; Essen et al., 2016). Over the recent years, these lower external costs—together with the expected increase in cargo flow in the upcoming decades—have induced a collective effort by governments, research institutions, and companies to generate a modal shift from road-based to waterway-based transport. For example, the European Commission has set the ambitious goal to push 30% of road freight transport (tkm), longer than 300 km, to rail and water-borne transport between 2011 and 2030, and 50% by 2050 (Kallas, 2011).

Today, most novel infrastructural and technological IWT developments focus on large waterways of type CEMT III–V (E. Verberght, 2019). Moreover, over the last few decades, cargo transport *via* smaller waterways—which enable connections to deeper parts of the European hinterland, and facilitates integration into the synchromodal transport network—decreased considerably (Tavasszy et al., 2015). This decrease can be partially attributed to: higher relative crew cost for smaller vessels, a lack of technological improvements, inadequate smaller waterway maintenance, and negative investment climate in the sector (Essen et al., 2016; Sys and Vanelslander, 2011).

Various local and European projects and developments contribute(d) to re-discovering the full capacity of the inland waterways, as well as to systems-of-systems automation and coordination. Two novel smaller vessel concepts (CEMT I–II) were recently introduced and constructed (The European Commission, 2019; Verberght, 2019) which exhibit a higher automation potential compared to older vessels (Peeters, 2021). Furthermore, the AVATAR (2021) project aims to develop urban vessels that are capable of navigating small rivers and canals in a highly automated manner. In addition, the European (IW-NET, 2021) and AUTOBarge (2021) projects investigate the integration of different automated vessels in the so-called “experimental living labs.”

Highly automated vessels and corresponding applications, as well as remote control and monitoring services, must integrate many tasks and motions at the same time. Moreover, they need to account for physical disturbances, and dynamically allocate software resources available within a particular environment (Bruyninckx, 2021). To enable these higher levels of automation for inland cargo vessels and their associated shoreside infrastructure, new IWT developments will need to incorporate *shared* situational awareness, and define, or extend formal knowledge systems accordingly (Peeters, 2021). In this regard, new developments can substantially benefit from a set of semantic world models, as part of this knowledge system, since it would allow co-operating (sub)systems to unambiguously interact with each other, as to understand environmental context or situation in which they operate. These models contribute to making safe, explainable, and resource-efficient control decisions.

### 1.1 Related Work and Developments

#### 1.1.1 Situational Awareness for Vessels and Operators

Argüelles et al., (2021) compiled a list of the main reported causes for ship collisions involving human errors. The authors argue, in compliance with findings in Sotiralis et al., (2016); Ung (2019); Weng et al., (2019); Yildirim et al., (2019); Huang et al., (2020), that the main causes for such collisions are: failure to take early action^1^, misinterpretation of collaborative regulations (COLREGs), and lacking communication between On-board Officers in charge of the Navigational Watch (OONWs)^2^. In other words, a lack of situational awareness (SA) for vessels and their operators (whether local or remote) lies at the root of these causes.

Then, Argüelles et al., (2021) proceed to investigate how to decrease collision risks, with a focus on low-level vessel-to-vessel communication, and dialogues. That is, by means of onboard Programmable Logic Controllers (PLCs)—in dialogue with OONWs—using the onboard AIS for vessel-to-vessel communication. Furthermore, as no standard for collision avoidance messages exists (IMO, 2001). Argüelles et al., (2021) proposes predefined messages and associated pictograms.

These developments emphasise the need for clear, explainable knowledge systems that can significantly enhance interactions between vessels, and between other related systems. In particular, the unambiguous application of COLREGs that are active in a certain situation, and the resulting actions/manoeuvres that need to be conducted, either implied or negotiated. As such, *Reconfigurable* and *explainable* ship domains can become a key communication and *visualisation* tool towards communication and visualisation, as well as reasoning.

#### 1.1.2 Ship Domains

The term “ship domains” most likely originated from Fuji and Tanaka (1971), whom defined it as a two-dimensional ellipsoid surrounding a ship—which other ships must avoid. Since then, many definitions of ship domains have occurred. In their review study, Szlapczynski and Szlapczynska (2017) distinguished three—partially overlapping—ship domain groups based on: 1) theoretical analyses; 2) expert knowledge; and 3) empirical methods. They concluded that the factors that the ship domains take into account are usually more meaningful than the exact domain shapes themselves, although the latter are often more emphasised in the literature. In addition, they noted that while many collision avoidance studies refer to ship domains, not many of them actually use these ship domains (Szlapczynski and Szlapczynska, 2017) in an operational context.

It should be noted that more context has been added to ship domain models by tuning domains for different geographical regions Hansen et al., (2013) and Wang and Chin (2016) and for different navigational situations. For example, as related to navigational situations, Liu et al., (2016) differentiated ship domains for the following four situations: 1) navigating along the channel; 2) crossing the channel; 3) another flow joining; and 4) turning. This paper focusses on adding the higher-order^3^ often symbolic, relations of the present situation and its context by explaining: *why* a ship domain was used, *what* it represents in this context, *how* the domains were computed, and *how* shared information can be interpreted by other entities.

#### 1.1.3 Semantic World Models for Maps and Robots

This work uses the term “world model” for any formal representation of the world, as part of the knowledge representation of reality and the real world, and conforms to the modelling policies described in Bruyninckx (2021), that, among other things, give the so-called open-world assumption a place in the policy of making models.

Semantic knowledge, i.e., formalised knowledge about objects, motion, tasks, events, and relations in the robot’s environment, can help both robots and human operators to perform specific tasks more effectively, and more importantly, allow for more enhanced systems-of-systems automation and coordination (Bruyninckx, 2021). A map where its features, in addition to spatial information about the environment, are mapped to entities of known classes, and, furthermore, that knowledge about entities is available for reasoning in some knowledge base with an associated reasoning engine, is called a semantic map (Nüchter and Hertzberg, 2008; Bakillah et al., 2013; Landsiedel et al., 2017).

Maps used by robots and vessel operators, both remote and onboard, typically consist of representation based on metrical and/or topological data structures. Additionally, these maps can be extended with sensor-specific data, for instance, pointclouds from perceptive sensors, possibly with additional information about texture. However, these representations do not take information about the objects and their properties into account, nor do they incorporate relations between specific objects and their environment, or a specific operational context (Lang et al., 2014).

### 1.2 Research Objectives

This study proposes, applies and experimentally verifies a set of semantic *world models* for maps and vessels. As such, it is investigated *how* these models can enable 1) the dynamic—context or situation-based—adaptation of ship domains, and 2) the unambiguous representation of entities in a local, semantic map.

The semantic descriptions in these world models, for instance of the geometry of features, can provide knowledge of where objects are with respect to each other in space and time. Position and motion of entities are always relative. Therefore, this work aims to model position and motion not as a property of an entity, but as a property of the relation between two entities. Consequently, such relations serve as the foundation for the dynamic world-model relations investigated in this paper, for example, the interactions between the local map and the vessel.

The following world-model relations will be handled explicitly or implicitly: 1) geometry–geometry: the representations of the shapes of objects and robots, and how they shape the motion constraints between objects, 2) geometry–perception: the representations of how properties of objects are detectable in sensor data, 3) geometry–motion: the representations of how properties of objects are targets of the motions of the robot, and 4) geometry–task: the representations of actual, desired, hypothesised, etc. states of the world, depending on the task requirements.

Throughout the experiments in Section 3.3.3, the following list of sub-objectives are dealt with:

• Define (higher-order) relations with (meta) models of the sensor subsystem, as part of the body model, that influence the geometric representation of the ship domains, for example, based on a geometry–geometry relation (Section 4.1), or a geometry–perception relation (Section 4.6).
• Enable dynamic configuration and adaptation of ship domains by introducing geometry–motion relations with vessel body entities, and geometry–geometry relations with map entities (Section 4.2 and Section 4.4, resp.).
• Allow for dynamic adaptations of both the hydrodynamic model and the vessel’s ship domains, by defining a descriptive^4^ model related to the vessel’s hydrodynamic characteristics, and adequate relations with other vessel subsystems (Section 4.3).
• Extend a local map with additional semantic features and metadata, to allow for unambiguous using and sharing of this map by other actors (Section 4.5).
• Integrate additional geometric entities at runtime that can help vessels and human operators to correctly interpret and automatically comply to COLREG rules in a particular situation (Section 4.6 and Section 4.7).

## 2 Materials

The main materials used throughout this work consist of four parts: 1) the semantic concepts for geometric world model compositions, handled by Section 2.1, 2) the research vessel named “the Cogge” (Peeters et al., 2020b) which relates to the body model, detailed in Section 2.2, 3) the (Inland) Electronic Navigational Chart objects as static features which relate to the map, discussed in Section 2.3, and 4) the hydrodynamic model, listed in Section 2.4 which, to some extent describes the physical connection between the vessel and the world (i.e., relating concepts of Section 2.2 and Section 2.3).

### 2.1 Semantic Concepts for Geometric World Model Compositions

The main semantic concepts for geometries in this work are 1) the entities of points, vectors, line segments, and polygons, 2) the relation of a map as an ordered or unordered set of geometric entities, 3) the relations of instantaneous motion of the vessel, and of its ship domains, and 4) the rigid body as a constraint on these motion relations. This work adopts a number of policies described in (Bruyninckx, 2021) to provide Semantic_IDs and relations to the entities described above. Consequently, this work uses the following two unique identifiers for a Semantic_ID ^5^:

• ID (“model ID”): a unique identifier with which the entity can be referred to, unambiguously, in another part of this model, or in other models.
• {MID} (“meta model ID”): a (set of) unique identifier(s) that each refer to a meta model, that is, a model in which the constraints are defined that describe the well-formedness of those entities and relations that the model uses from that particular meta model.

In Bruyninckx (2021), it is suggested all geometric entities are compositions of the Point model, whereas this work makes a distinction between two geometric feature compositions. The first one involves dynamically composed geometries from external datasets such as (I)ENC or OpenStreetMap, which—on the application level—can be queried from a database, and where the results can contain any of the types defined by the GeoJSON data format^6^. These features merely have a Semantic_ID and relations of the whole entity, and not on every coordinate. The second one includes geometries that are composed from a Point model. The latter can be desirable in a robotics context, as most sensors in robotics measure points, and not lines, planes, or bodies. Moreover, the concept of uncertainty is well-defined for points, but not for lines, planes, or bodies.

A mereo-logical^7^ model of a Point in two-dimensional Euclidean space (E2), with Semantic_ID metadata, is referred to as {Point: aPoint} (with “aPoint” the name for this Point entity), and represented in full as follows:

Listing 1:
Mereo-logical model of a Point

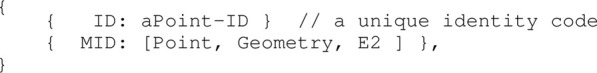

Other relevant representations of geometric entities include:

• Vector: an ordered list of two Point entities that adds three constraints: 1) the ordering that gives an orientation to the Vector, 2) that list contains exactly two members (and not all the points on the line in between), and 3) the two Points in the list are different.
• Simplex: (in a 2D space) an ordered list of two non-colinear Vectors, with the constraint that both have the same start point.
• LineString (or polyline): as an ordered set of points. It is equivalent to an ordered list of Vectors, with the constraints that 1) the start point of one Vector is the end point of the previous Vector in the ordered set, 2) each Point belongs to exactly two Vectors, except for the start point of the first Vector and the end point of the last Vector.
• Polygon: similar, with the extra constraint relation that the two not yet connected start and end Points must now coincide.
• Frame: it adds two metric constraints to the Simplex entity: 1) the length of each Line segment is the unity length, and 2) the Line segments are perpendicular (or orthogonal). The orientation of a Frame is typically an essential attribute, because of its use to represent coordinates.

Models of these entities are shown in Listing 2.

Listing 2:
Models for geometric entities

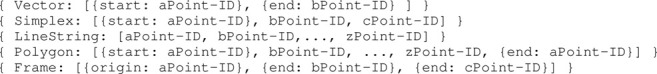

When no explicit name of an entity is known, or defined, this work uses the notation aModel_Name (prepending “a,” “b,” etc., to the model name). The associated IDs of an entity are named with the name of the entity, added with the postfix “-ID.”

### 2.2 Cogge: An Unmanned Inland Cargo Vessel

**FIGURE 1:**
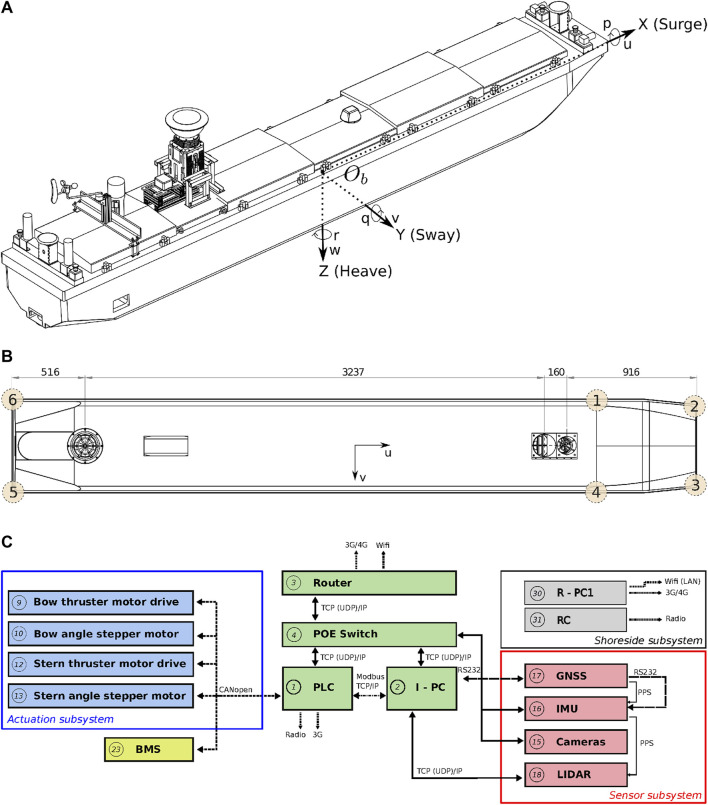
Geometry of the *Cogge*: **(A)** full geometry and reference frames, **(B)** bottom view of the hull with longitudinal dimensions, and **(C)** main components and communication links (as a single vessel). Panel **(A)** and **(B)** were modified from Peeters et al., (2020c), and **(C)** reproduced from Peeters et al., (2020d).

Peeters et al., (2020b) constructed a scale model unmanned inland cargo vessel to investigate the automation potential of its real-size counterpart. Figure 1 summarises the relevant technical details of this vessel, named the *Cogge*: (a) shows its three dimensional geometry, together with its body-fixed reference frame, (b) draws a two dimensional bottom view of the *Cogge*, which illustrates the position of its actuation system, and (c) provides a communication scheme of the main onboard components. A video of this vessel in operation is included in the Supplementary Video (S0).

### 2.3 The Inland Navigational Charts ((I)ENC)

Navigational charts for vessel operators, vessel autonomy systems, remote control operators, and remote monitoring services, use (I)ENC charts as the *de facto* standard dataset. These charts are typically displayed, and augmented with sensor data, in an (Inland) Electronic Chart Display and Information Systems (ECDIS). These chart displays have proven to be an indispensable navigational aid for operators. It already contains a standardised set of object models, including a limited set of semantic tags in the form of attributes. However, they lack adequate semantics for situational-aware reasoning in highly automated environments, and are not dynamic. For instance, they are—depending on the administrator—only updated on a yearly basis, there are no accuracy indications of chart features, additional (dynamic) data such as RADAR pointclouds or data from Automatic Identification Systems (AIS) is added as a separate, *independent* layer, and there are no policies in play for dynamically defining, updating, and adding entities and relations between these objects. This means, for the purpose of this work, that the (I)ENC charts serve as the static feature base of the map, which is extended with additional relations and semantic information, and context-dependent functionality at runtime.

Tsou (2016) uses ECDIS as a platform for constructing a collision-avoidance decision support system. The ENC features and their corresponding geodetic coordinates, together with data from sensors such as AIS, integrated with the ECDIS, are used to predict areas of danger within a specific environment, and to plan the optimal route accordingly. This implies the need for rather complex geographical computations, whereas considering the ranges (up to one or several kilometres) to which these collision avoidance schemes apply, a local Cartesian reference systems, and corresponding map features with local relative coordinates, could significantly reduce the reasoning complexity, and thus reduce the need for computational resources. Moreover, it allows for a more straightforward integration with other software components, for instance towards control and coordination of the robot.

**FIGURE 2:**
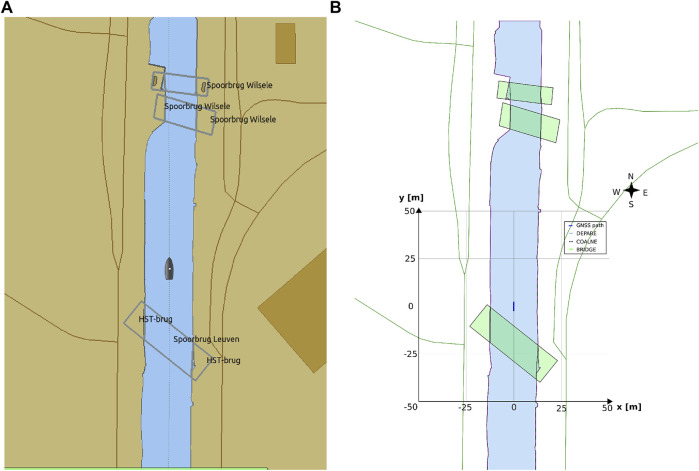
OpenCPN ECDIS display, with global ENC features projected onto screen **(A)**, versus a local map **(B)** with features in local ENU coordinates, generated from global ENC dataset, displayed as a vector image (in SVG).

Global maps and reasoning based on (projected) geographic features are relevant for mid- and long-term planning and decision making, that is, within a timeframe of minutes up to days. For these purposes, several established tools are available, not in the least spatial indexing and querying of features in a database (often with a GIS extension) (Bakillah et al., 2013). However, tasks that require short-term (seconds up to minutes) reasoning and decision making, knowledge about the dynamic body models of systems, and/or relative information about sensor data or the operational environment, could benefit substantially from interacting with local maps and features. This is especially true in highly dynamic environments, which are the focus of this paper. As such, the geometric features and models used in this paper consist of dynamically generated, local coordinates. Features related to a vessel’s navigation environment, mainly constructed from (external) global coordinates, contain local tangent plane coordinates (East-North-Up). Other local frames that are part of the world model, such as body-fixed sensor frames, will include at least one symbolic relation with a point on the local reference frame. Figure 2 shows both the ECDIS map and the corresponding local map used throughout this work.

### 2.4 Hydrodynamic Modelling

The rigid-body kinetics of a vessel can be written in a vectorial setting according to Fossen (1991):

$$M_{RB}\dot{\nu }+C_{RB}\left(\nu \right)\nu =\tau_{RB},$$ (1)

where ***M***_*RB*_ represents the rigid-body inertia matrix, ***C***_*RB*_ the rigid-body Coriolis and centripetal matrix, ***τ***_*RB*_ = [*X*,*Y*,*Z*,*K*,*M*,*N*]^*⊤*^ the vector of generalised forces, and ***ν*** = [*u*,*v*,*w*,*p*,*q*,*r*]^*⊤*^ the generalized velocity vector, when using the SNAME convention SNAME (1950). Here ***τ***_*RB*_ can be separated into:

$$\tau_{RB}=\tau_{hydrodynamic}+\tau_{hydrostatic}+\tau_{wind}+\tau_{waves}+\tau_{control}$$ (2)

In addition, one can rearrange the terms into the following vectorial setting Fossen (1994), Fossen and Fjellstad (1995):

$$\underset{\text{rigid}-\text{body}}{\underset{︸}{C_{RB}\left(\nu \right)\nu +M_{RB}\dot{\nu }}}+\underset{\text{hydrodynamic}}{\underset{︸}{C_{A}\left(\nu \right)\nu +D\left(\nu \right)\nu +M_{A}\dot{\nu }}}+\underset{\text{hydrostatic}}{\underset{︸}{g\left(\eta \right)+g_{0}}}=\tau_{control}+\tau_{wave}+\tau_{wind}(3)$$

with:

• ***M***_*RB*_(***ν***) and ***M***_*A*_(***ν***): system inertia matrices, (rigid body and added mass, resp.)
• ***C***_*RB*_(***ν***) and ***C***_*A*_(***ν***): Coriolis-centripetal matrices, (rigid body and added mass, resp.)
• ***D*** (***ν***): damping matrix
• ***g*** (***η***): vector of gravitational/buoyancy forces and moments
• ***g***_0_ vector used for pretrimming (ballast control)
• ***τ*** external forces (control, wind, and wave)

Here the modular matrix–vector notation can lever matrix properties such as symmetry, skew-symmetry, and positiveness of matrices Fossen (2011).

This study models the planar motions of the *Cogge*: ***ν*** = [*u*,*v*,*r*]^*⊤*^. Hence the impact of heave, roll, and pitch motions are neglected, together with their hydrostatic restoring forces, i.e., ***τ***_*hydrostatic*_ = 0. The vessel will operate under the Manoeuvring Theory framework Fossen (2011) assumptions, ***τ***_*wave*_ = 0, i.e., the (hydrodynamic) manoeuvring coefficients can be assumed to be frequency-independent, in calm water without current. Further assumptions include: a homogeneous mass distribution, the ur-plane symmetry of the vessel, i.e., *I*_*uv*_ = *I*_*vr*_ = 0, the origin of the body-frame (CO) positioned on the centre line of the vessel, i.e., *v*_*g*_ = 0, calculating the added mass terms in CO, and aligning the CO axes with the principal inertia axes of the vessel.

The damping matrix will be modelled with a linear and non-linear part: **D**(***ν***) = **D**_**L**_ + **D**_**N**_(***ν***). The linear damping components are important for the lower speed manoeuvres Fossen (2011). The nonlinear damping will be modelled by a quadratic surge resistance (Lewis and Resistance, 1989) for the surge motion, and by a cross-flow-drag model fitted Blanke (1981) to second-order modulus functions Fedyaevsky and Sobolev (1964) for the sway–yaw motions Fossen (2011). More precisely, a simplified form will be used Blanke (1981), normally intended for larger vessels, but providing sufficient parameters to capture the motions of the *Cogge* for the purpose of this study.

For the control forces vector, Peeters et al., (2020c) concluded that thruster models which neglect the vessel-speed-dependent thrust losses can still suffice to capture and describe the main hydrodynamic vessel behaviour in pure surge, sway, or yaw motion. Therefore, vessel-speed-independent thruster models will be used throughout this study. Furthermore, wind forces are modelled as seen in Fossen (2011). with associated wind coefficients as derived by (Isherwood, 1972). Under the abovementioned assumptions, Eq. 3 refines to:

$$\underset{\text{rigid}-\text{body}}{\underset{︸}{M_{RB}\dot{\nu }+C_{RB}\left(\nu \right)\nu }}+\underset{hydrodynamic}{\underset{︸}{M_{A}\dot{\nu }+C_{A}\left(\nu \right)\nu +D_{N}\left(\nu \right)\nu +D_{L}\nu +}}=\tau_{control}+\tau_{wind}$$ (4)

Supplementary Appendix A details the resulting full component model, and its identified or estimated coefficients can be found in Listing 19.

## 3 Methods

**FIGURE 3:**
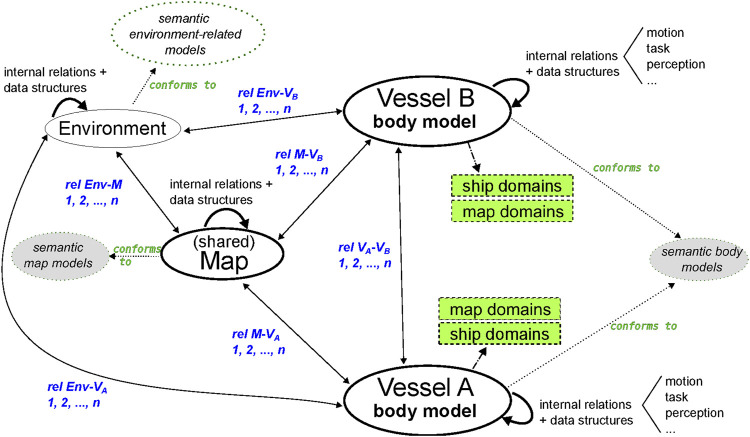
High-level structure for model-integration and relations discussed throughout this paper, applied to a situation with two interacting vessels. Relations (rel) in blue. Grey ellipses (models) and green rectangles (ship and map-domains) are the main focus areas of this paper.

As discussed in Section 1.2, this paper focusses on vessel-world interactions, employing internal and external world models, as related to inland waterway navigation along a channel. Section 3.1 and Section 3.2 describe, respectively, the internal (i.e., body) and external (i.e., map) world models. Section 3.3 illustrates such potential dynamic interactions, as well as maintained relations, between world models, by means of ship domains (SDs) and map domains (MDs). The high-level architecture depicted in Figure 3 gives an overview of the model-conform relations and entities discussed in this paper. While this figure includes only four entities (map, environment, two vessels), it should be noted that relations with other (external) entities can co-exist as well. The corresponding experiments mainly focus on semantic models (grey ellipses), as well as on context-dependent ship and map domains (green rectangles).

The models discussed here are represented using a JSON-*like* syntax^8^, and their main aim is to demonstrate a methodology of composing such models. As such, not every symbolic model and corresponding data structure(s) used in Section 3.3 is listed in full throughout this section. Furthermore, whenever a symbolic model does not provide additional insights on model compositions, it is *not* listed in this work. Its corresponding data structure, however, often *is* listed, that is, whenever this information is deemed relevant with respect to the experiments. While these data structures already provide a formal basis for situational-aware applications (see Section 3.3), these models are not complete or meant to be generic. Rather they serve the purpose of a starting point on which (external) input, test results, and other feedback can be provided towards further development (see the discussion of Section 5).

### 3.1 Semantic Internal World Model or Body Model

World models related to the vessel and its subsystems are part of the body model. Figure 1 already showed the main components of the research vessel the *Cogge*. The body models discussed here aim to provide adequate semantic information to enable subsequent reasoning, and connecting these models with the external world model, i.e., the map. In that sense, the following entities and relations are considered relevant to the body model:

•Vessel: represents the vessel (main entity), with relations to other entities described hereafter.
•Hull: Describes characteristics about the hull of a vessel, e.g., geometries.
•Hydrodynamics: hydrodynamic model structures and their parameters (to support context-driven dynamic configuration), as well as their relations to control tasks of the vessel.
•Actuation: vessel actuation model, with lower-level models for bow and stern (potentially more). Both bow and stern contain an explicit geometric relation with the Hull
•Sensors: the sensor subsystem, further divided into:
•Proprio-ceptive: GNSS and IMU sensors (for Cogge)
•Extero-ceptive: LiDAR and cameras (for Cogge)
•Carto-ceptive: available semantic maps for a vessel or service
•Communication: available communication channels, protocols, etc., divided into internal communication and external communication.

For example, AIS-related models are part of the Communication entity, however, a detailed specification of the communication entity and its sub-entities is outside the scope of this paper. Additionally, the existence of a set of (meta) meta models relevant to the IWT context is assumed, with the most generic meta model: {MID: Inland_Waterway_Transport}, providing domain-specific metadata for the models by means of the MID of the Semantic_ID.

#### 3.1.1 Vessel

Listing 3 provides a high-level symbolic model for IWT vessels, with a limited set of properties. Its ID is related to the MMSI property, as it already provides a unique, 9-digit identifier for a vessel.

Listing 3:
Iwt_Vessel_Name_Mmsi_Cemt model

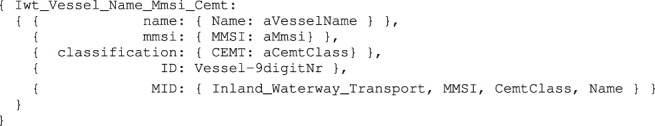

The meta-models Inland_Waterway_Transport, MMSI, CemtClass, and Name contain information about data types, description, units, among other things related to the attributes in the Iwt_Vessel model. A data structure model adds numerical values to the symbolic model properties, as an instance-of the Iwt_Vessel_Name_Mmsi_Cemt model:

Listing 4:
Iwt_Vessel_Name_Mmsi_Cemt_Data instance

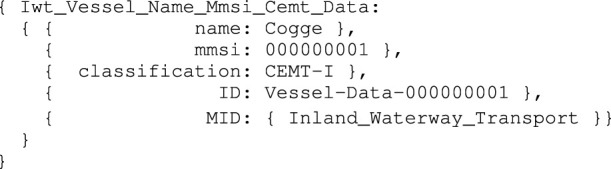

An Iwt_Vessel_Name_Mmsi_Cemt entity is in several relations. For example: it has-a Iwt_Hull_Base entity, and, inversely, the Iwt_Hull_Base is part-of (or belongs-to) an Iwt_Vessel_Name_Mmsi_Cemt entity.

#### 3.1.2 Hull

Similar to the above, a model for the vessel’s hull can be composed (i.e., Iwt_Hull_Base). It provides sufficient symbolic information on data types, units, etc. Note that this symbolic model is not provided explicitly, instead, Listing 5 defines the associated data structure model of the vessel’s hull:

Listing 5:
Iwt_Hull_Base_Data instance

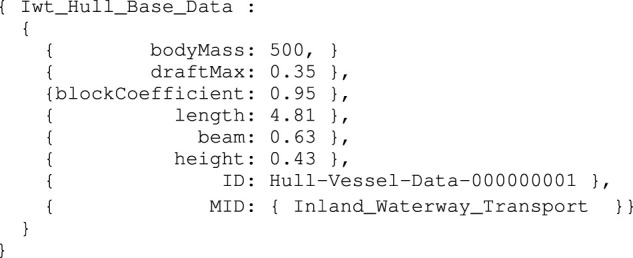

In Listing 6, a set of already defined mereo-logical Point entities is assumed, according to the model in Listing 1. These include a body-fixed origin *o*_*b*_: PaPnt0 (Pa refers to principle axis), together with PaPntU (on longitudinal vessel axis), PaPntV (on transversal vessel axis), and PaPntR (on normal axis). Moreover, the origin point PntCo is defined, and relates to a point of the vessel’s midship, at the height of the water line (this must be modelled as a constraint). In our case, the explicit relation exists between PaPnt0 and PntCo, that is, the centroid of the vessel and the body-fixed origin have the same absolute position. The associated model for the vessel’s principal axes, also used in Section 3.1.3, is defined in 6.

Listing 6:
Iwt_Vessel_Principal_Axes_Frame model

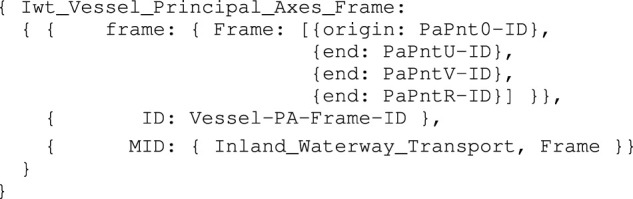

To enhance in-model reference clarity, an entity that conforms to the Iwt_Vessel_Principal_Axes_Frame model is hereafter called PA, which is part-of the Iwt_Vessel_Name_Mmsi_Cemt entity. Other relations with this PA are discussed in Section 3.2. Assuming the points PntGeom1, …PntGeom6, are also symbolically defined, corresponding to the top view of the vessel of the vessel in Figure 1, a Coordinate model is constructed for these points. This model adds 1) numerical values to the earlier defined mereo-logical models defined already, as a position relative to an previously defined frame, and 2) the semantic tags for identifying the correct interpretation of those values. For the first point entity PntGeom1, the Coordinate model is defined in Listing 7.

Listing 7:
Coordinate_Point_Point_Frame_Meter_Data instance

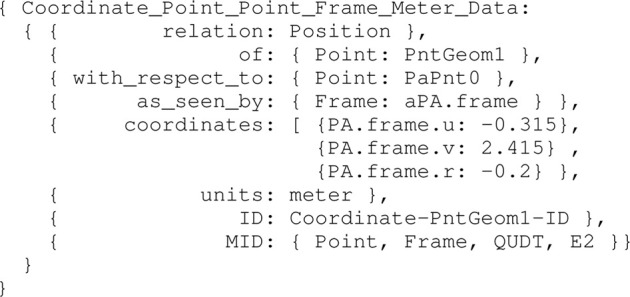

Note that the meta model QUDT is used here as well, including abstract representations of Quantity, Unit, Dimension, and (data) Type. The same Coordinate models are constructed for the other points in, identified in the 2D top view. By means of combining the aforementioned Point entities, various geometries can be composed. Since this work explicitly uses the 2D top view geometry of the vessel’s hull in the semantic map (see Section 3.2), as well as for the ship domains (see Section 3.3.1), a model is constructed for this 2D top view, as a Projection relation:

Listing 8:
Multiview_Projection_Entity_Plane_Type_Result model

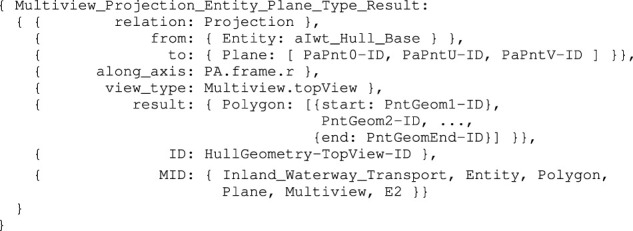

The along_axis property is to some extent redundant to the Plane of the vessel. The Plane is defined as a join of three Points, even though other definitions are possible as well, for instance, a join of intersecting Lines.

#### 3.1.3 Hydrodynamics

The hydrodynamic behaviour of a vessel is not only relevant to simulation or control software, but is also relevant to (higher level) reasoning tasks and visualising ship domains. It is important to note that different behavioral models of a vessel can be appropriate to different situations. Even though a single model can capture behavior appropriate to all situations, such a “one-size-fits-all” hydrodynamical model is often overly complex for the situation at hand. Hence, considering computational efficiency, accuracy, degrees of freedom, observability, controllability, available identified coefficients, among many other factors, can motivate context-related simplifications.

Therefore, dynamic, context-dependent composability is a desired property of hydrodynamic models. In this work, a set of meta models are assumed to be available, for instance, Hydro_Equation_Manoeuvring_F is used as the meta model for Eq. 3. Other meta models relate to Fossen (2011). In this work, entities that conform to such models are here as follows: aHydro_States_F, aHydro_Ext_Forces_F, and aHydro_Coefficients_F.

First, three additional body-fixed reference points are defined: PntCg (center of gravity), PntCB (center of buoyancy), and PntCf (center of flotation). Coordinate models as seen in Listing 7 can be used to represent these points. For our vessel, the Cogge, it is a reasonable assumption that all three Points have the exact absolute position as PaPnt0. The model used in this study, as shown in Listing 9 considers three degrees of freedom (surge, sway, yaw).

Listing 9:
Iwt_Hydrodynamic_3DOF_States_Forces_Coef model

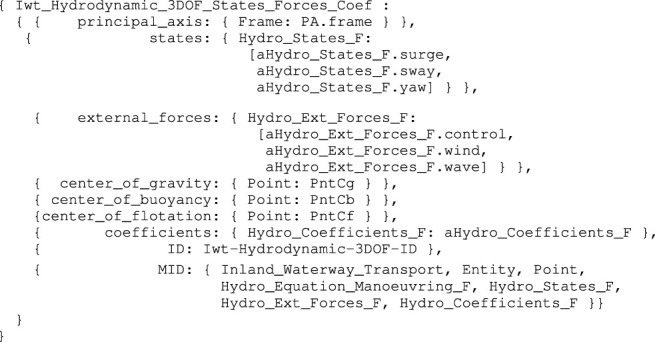

Corresponding data structures for the states and forces can be constructed as an instance-of the respective embedded symbolic models of Listing 9. Similarly, a data structure for the coefficients is constructed, as an instance-of Hydro_Coefficients_F. The values for these coefficients, used in the data structure, can be found in Supplementary Appendix A1.

With respect to the behaviour of the real vessel, and as discussed in Section 3.3.3, a set of constraint relations are defined as well, mostly relating to the instantaneous motion of the vessel. They can apply to one or several matrix components as seen in Supplementary Appendix A1. One example is a switch between linear damping (*D*_*L*_), and linear + non-linear damping (*D*_*N*_ + *D*_*L*_) when the vessel exceeds some velocity threshold. This threshold was determined experimentally, and can be modelled to a constraint. Both the velocity and the threshold constraint are modelled as relations, respectively in Listing 10 and Listing 11.

Listing 10:
Coordinate_Velocity_Point_Frame_Meter_Data instance

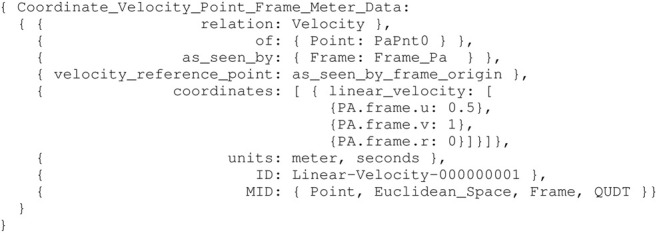

Consider an entity aVel conform to the model in Listing 10, then the threshold constraint model can be defined as follows:

Listing 11:
Iwt_Hydrodynamic_Constraint_Entity_Velocity_ Data instance

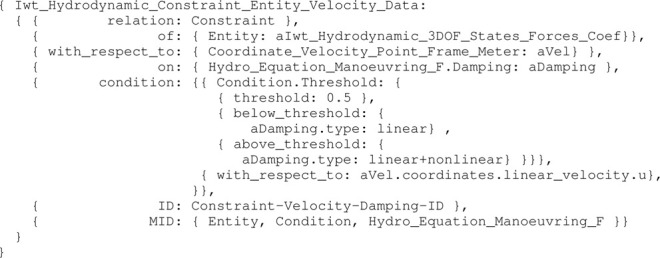

The hydrodynamic constraint model listed in Listing 11 provides sufficient information to switch programatically between linear and linear+non-linear damping. Note that a similar constraint is included for velocity in the v-direction, and a third constraint relation between both of these to determine the damping outcome unambiguously. Many other constraint relations can be added to a hydrodynamical model (e.g., to its coefficients), to support specific behaviours of or tasks performed by a vessel. A limited set of such constraints is illustrated in the experiments in Section 3.3.

A different geometry–motion relation used for the ship domains of Section 3.3.1, related to the hydrodynamic behaviour of the vessel, consists of deceleration-related (third-order polynomial) coefficients, both to model 1) natural deceleration, i.e., the distance travelled by the vessel when no longer actuating (natural speed decay), and 2) actuated deceleration, i.e., the distance travelled by countering motion through reverse actuation commands for braking. Assuming the symbolic model, Iwt_Hydrodynamic_Entity_Deacceleration_Coef is already defined, as part-of a Iwt_Vessel_Name_Mmsi_Cemt entity, the corresponding data structure, as an instance-of this symbolic formalism, then becomes:

Listing 12:
Iwt_Hydrodynamic_Entity_Deacceleration_Coef_Data instance

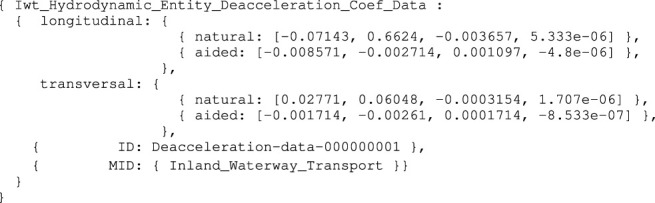

Note that the Iwt_Hydrodynamic_Entity_Deacceleration_ Coef model contains all the necessary information, including appropriate meta models, to correctly interpret the data structure.

#### 3.1.4 Actuation

Actuation, and the ability to reason about a vessel’s actuation system, is essential for (remote) vessel operators, and for control software, to safely and effectively navigate a vessel. It can provide a minimal basis for mapping control inputs from joysticks in remote control centres to actuation commands of the vessel, and for mapping actuation commands to forces in the hydrodynamical model. Moreover, monitoring services should be able to identify, and potentially anticipate on, (electro-)mechanical failures as accurately as possible, especially when a vessel faces a handicap in performing its tasks.

Here too the assumption is made that a model Iwt_Actuation_Entity_Bow_Stern, with a conform entity as a part-of aIwt_Vessel_Name_Mmsi_Cemt already exists, containing all necessary metadata for unambiguously interpreting the data structure. For example, a specific (constraint) relation exists between the dimensions of the actuation system and the vessel geometry defined earlier. This data structure then becomes:

Listing 13:
Iwt_Actuation_Entity_Bow_Stern_Data instance

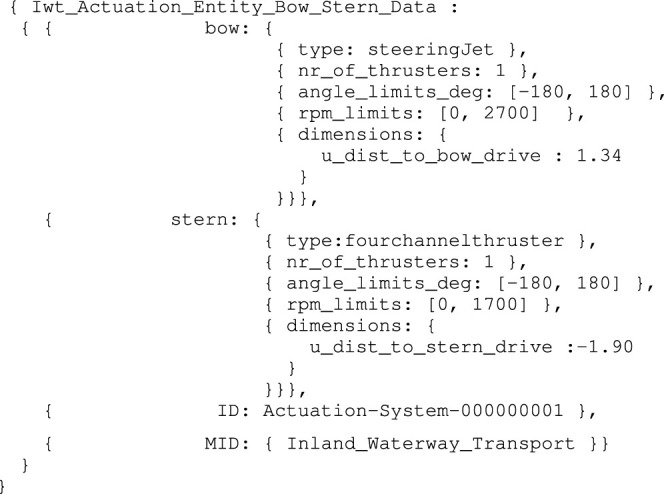

Note that other information, for instance on the correct interpretation of the lookup-table for mapping drive system input commands to propulsion forces, can also be included as a part-of the actuation system model.

#### 3.1.5 Sensors

Similarly to the actuation subsystem, the sensor subsystem also strongly relates to control and decision making, but from an information source point of view. In the following, explicit relations with ship domains, and the semantic map are introduced, based on sensor information.

As introduced earlier, a distinction is made between the following subsystems:

• Proprio-ceptive sensors: sensors to obtain information related to the vessel’s own body model, e.g., GNSS, IMU, etc.
• Extero-ceptive sensors: sensors related to the vessel’s perception, and thus related to extending world models with objects/entities/actors in the vessel’s environment, e.g., LiDAR, cameras, ultrasound distance sensors, etc.
• Carto-ceptive sensors: maps that are available to the vessel, also related to extending the vessel’s world model with objects/entities/actors in the vessel’s environment, that are often not directly perceivable by the robot’s extero-ceptive sensors, or are difficult to perceive.

For navigation, vessels typically use information from either a map, or from their perception sensors, as part of their world model to perform specific tasks. However, in order to use both of them together, switch between them as driven by situations and context, or in order to validate one another, additional (meta) information about the data is crucial. Detecting known map-landmarks in the perception sensor data, or vice versa, is only possible if these landmarks are unambiguously identifiable, and thus have appropriate semantic tags.

A complete set of models for the sensor subsystems is beyond the scope of this work, however, current information used for the experiments in Section 3.3, are interpreted according to the model in Listing 14, which obviously is an instance-of aIwt_Vessel_Name_Mmsi_Cemt. Also note that further decoupling of this model might be desired, however, this was not necessary for the experiments in Section 3.3.3.

Listing 14:
Iwt_Sensor_Proprio_Extero_Carto model

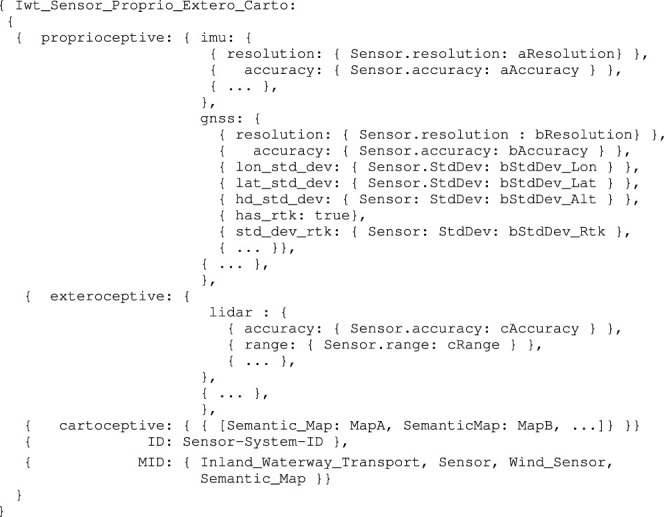

The cartoceptive value here is a set of semantic maps. The map discussed in Section 3.2 could be one of them.

### 3.2 Semantic External World Model or Map Model

#### 3.2.1 Map Initialisation

As stated in Section 2.3, local tangent plane coordinates (ENU) are used as a geographic reference system for the 2D maps presented in this work. A local map will be generated at runtime, on a specific timestamp, based on a specific situation, by some entity. Note that in the context of this paper, maps are generated by the vessel entity. The body-fixed origin *o*_*b*_ of the vessel, coinciding with a Point on the vessel that is tangent to the waterline, is used as origin of the local map. Assuming the Point EnuPnt0 is defined, we get:

Listing 15:
Iwt_Map_Entity_Point_Time model

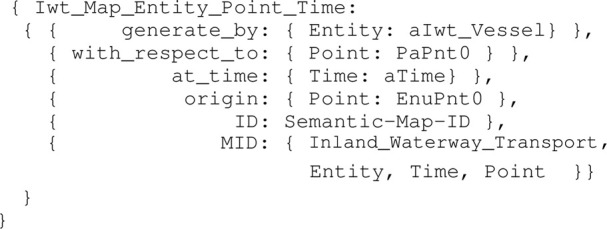

Moreover, assume a defined reference frame model Geodetic_Frame_Enu, as a composition of the points EnuPnt0, EnuPntX, EnuPntY, and EnuPntZ. Then, the map entity conforming to Iwt_Map_Entity_Point_Time has a Geodetic_Frame_Enu entity. The coordinate data structure for the origin EnuPnt0 is shown in Listing 16.

Listing 16:
Coordinate_Point_Entity_Frame_Enu_Data instance

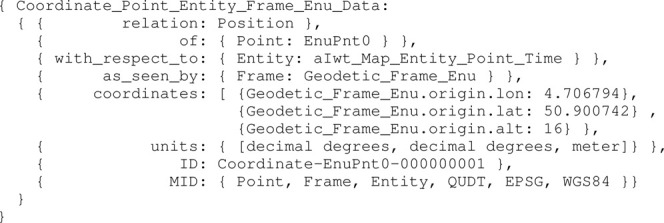

Correct interpretation of this origin Point is absolutely critical, and has several additional geometry relations with vessel entities. When the map is shared between such entities, or with remote control centres, a necessary set of meta data allows for correct interpretations and model-to-model transformations, as every entity or service can have their own sets of conventions and compositions.

The conversion from geographic features, containing geodetic coordinates in the EPSG:4326 reference system, to local ENU coordinates, is a two stage process, involving 1) to convert geodetic coordinates to ECEF coordinates, and 2) to convert ECEF coordinates to local ENU coordinates. The first step 1) is performed by using the following equations, with latitude *ϕ*, longitude *λ*, and height *h*:

$$X=\left(N\left(\phi \right)+h\right)\cos\phi \cos\lambda$$ (5)

$$Y=\left(N\left(\phi \right)+h\right)\cos\phi \sin\lambda$$ (6)

$$Z=\left(\frac{b^{2}}{a^{2}}N\left(\phi \right)+h\right)\sin\phi$$ (7)

where

$$N\left(\phi \right)=\frac{a}{\sqrt{1-e^{2}\sin^{2}\phi }},$$ (8)

and *a* and *b* are the equatorial radius semi-major axis and the polar radius semi-minor axis, respectively. Also note that here $e^{2}=1-\frac{b^{2}}{a^{2}}$ is the square of the first eccentricity of the ellipsoid. The prime vertical radius of curvature, *N*(*ϕ*), is the distance from the surface to the Z-axis along the ellipsoid normal. For the second step 2), given a local reference point $\left\{X_{r},\;Y_{r},\;Z_{r}\right\}$ , and an object at $\left\{X_{p},\;Y_{p},\;Z_{p}\right\}$ , then the vector pointing from the reference point to the object in the ENU frame is:

$$\left[\begin{array}{c} x\\ y\\ z \end{array}\right]=\left[\begin{array}{ccc} -\sin\lambda_{r} & \cos\lambda_{r} & 0\\ -\sin\phi_{r}\cos\lambda_{r} & -\sin\phi_{r}\sin\lambda_{r} & \cos\phi_{r}\\ \cos\phi_{r}\cos\lambda_{r} & \cos\phi_{r}\sin\lambda_{r} & \sin\phi_{r} \end{array}\right]\left[\begin{array}{c} X_{p}-X_{r}\\ Y_{p}-Y_{r}\\ Z_{p}-Z_{r} \end{array}\right]$$ (9)

The envelope of the local map can be determined by the user, the situation, or by the application.

**FIGURE 4:**
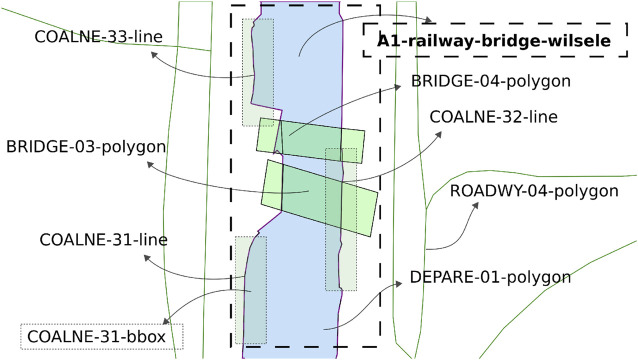
Part of local map from Figure 2B with Symbolic_IDs and additional metadata.

At the timestamp the map is generated, additional metadata is added to the features fetched from external sources, which allows for more efficient querying, sharing, and context negotiation at runtime. An example is illustrated in Figure 4. In that example, the following semantic tags are allocated:

• navbounds: symbolic tag allocated to (I)ENC features that represent navigation boundaries, here corresponding to COALNE (coastline, or shoreline) and DEPARE (depth area, i.e., water area with depth between a defined range of values) features (see also Figure 7).
• enc-feature-X: relation with corresponding (I)ENC feature, also connecting the already existing tags such as object names (OBJNAM), general feature info (INFORM), and information about the visibility of objects to a certain context. For instance, the CONRAD attribute, which provides information on the conspicuousness of the feature for radars, e.g., whether this returns a strong radar echo. Note that this can then be connected with the model of the sensor subsystem, as given in Listing 14.

Furthermore, the following geometries and corresponding relations are defined:

• A1: sub-area in the map containing all features within a certain range (property) of BRIDGE-04, that is, the railway bridge around the research area in Leuven, Belgium. Similary, this is done for locks, terminal, and any other critical regions. Note that these areas are computed at the initialisation of a map, using (geo)spatial computations on the features inside the global map.
• FEATURE-NR-bbox: bounding-box of a feature in the map, which can be used, for example, to model constraint relations like Intersections.

Furthermore, a set of attributes is given to all features. For example, number_of_points, or feature_type, as well as the set of local coordinates. The number in the feature IDs is also a property of each feature, and the number sequence is related to a predefined meta-model.

It should be noted that multiple maps can be generated, initialised at different timestamps and/or by various entities (which can in turn be related to one another).

#### 3.2.2 Ship Position on the Map

A coordinate model for Position of the vessel as a relation between the Point geometry PaPnt0-ID (body-fixed origin of vessel) and the Geodetic_Frame_Enu (Frame geometry) of the local map, is constructed as follows:

Listing 17:
Coordinate_Point_Entity_Frame_Meter data instance

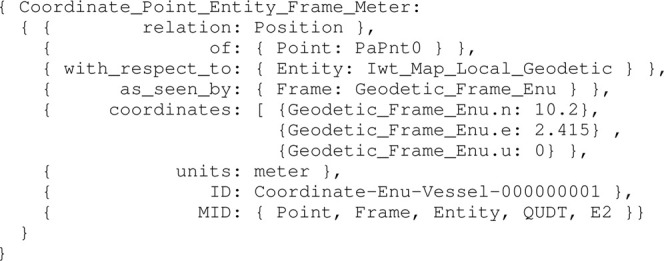

**FIGURE 5:**
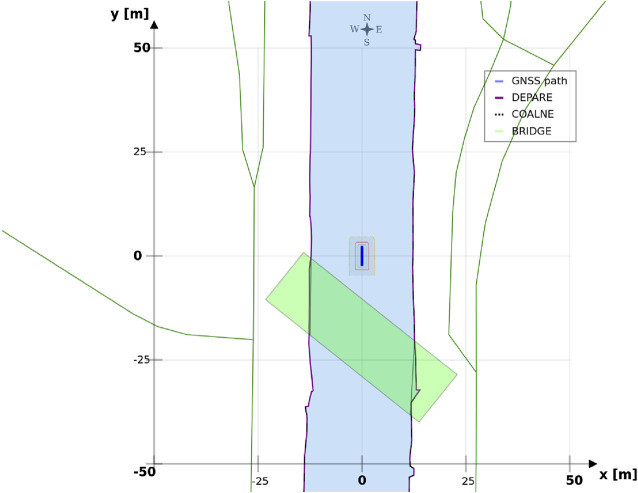
Vessel, represented as a blue polygon and surrounded by ship domains, in local ENU frame, at position (0,0).

The position [0, 0] of the vessel in the local ENU reference system, is illustrated in Figure 5.

Similar to the ship Position relation model above, models for velocity, acceleration, etc., can be constructed. Generally speaking, motion has three parts: the Position relation, and the relations of Velocity and Acceleration that represent the first- and second-order derivatives in time of the Position relation. Because of this relation model, the same geometric entity can have several Positions and Motions at the same time: one for each other entity involved in a Position or Motion relation, and thus in this case, relative to a specific feature entity in the semantic map.

Furthermore, the top view Projection of the vessel hull is used in the map, as seen in Listing 8, with the following two constraint relations:


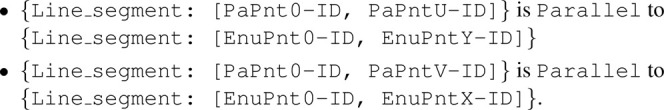


### 3.3 Semantic Dynamic Models for IWT

This work uses ship domains (SD) and map domains (MD) to illustrate relations that are relevant within a situation. The adjective *dynamic* denotes the ability to update these sets of model-conform entities and relations at runtime. A modelling approach for these domains is briefly discussed in Section 3.3.1 and Section 3.3.2. Subsequently, these models are used in the range of experiments of Section 3.3.3, where situations are considered that require dynamic behaviour. Note that corresponding results are covered in Section 4.

#### 3.3.1 Ship Domains

Dynamic, context-dependent ship domains have relations with the body model of the vessel, and with the semantic map. These SDs are the main focus throughout the experiments in Section 3.3.3, as they allow for situational-aware reacting of the application, and subsequent visualisation, based on the integration of the internal and external world models. The ship domain geometries contain relations with (a combination of) the Motion, Task, Perception, and Map of the robot.

**FIGURE 6:**
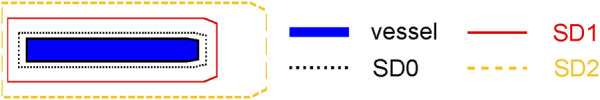
Example configuration of vessel ship domains.

In the recently finished hull-to-hull (H2H) project, experiments were conducted with the explicit use of uncertainty and proximity vessel zones as a navigational aid for remote operators SINTEF (2020); Kotzé et al., (2019). In accordance to this project, this work adopts a similar three-level domain taxonomy, and subsequently focusses on the composability of the SDs, based on earlier defined relations. The three domains, illustrated in Figure 6, are defined as follows:

• **SD0** (“…”, black dotted line): uncertainty zone, discussed in Experiment 1.
• **SD1** (“—”, red line): sometimes referred to as the danger zone, as it is typically related to the required breaking distance (Motion) of the vessel, as seen in Experiment 2–Experiment 5.
• **SD2** (“- -”, orange dashed line): the geometry of this zone is highly dependent on the situation, and various representations or paramterisations exist depending on its (set of) relation(s), illustrated in Experiment 2–Experiment 7.

This work advocates to have at least one, fundamental, axiomatic, ship domain “SD0” as part of its world model, which connects the vessel with the map, taking into account the knowledge of the uncertainty of the position associated with the symbolic connection point, and the orientation of the vessel. This information is considered essential to enable higher levels of autonomy. Moreover, two additional zones (“**SD1**” and “**SD2**”) make sense from the perspective of a human interpreter, however, as discussed before, a vessel can interpret several domain compositions and relations simultaneously. As such, more (sets of) ship domains can be added at all times, given they have semantic relations that allow for unambiguous interpretation of these domains.

Consider the mereo-logical models of these three ship domains, and their conforming entities defined as: SD0, **SD1** , and **SD2** . Next, the geometries of these ship domains and their relations need to be defined. For instance, for SD0, this can be a two-dimensional affine transformation of the top view hull geometry entity. A model for this **SD0** is shown in Listing 18:

Listing 18:
Iwt_Ship_Domain_A2_Scale_Rotate_Skew_Translate model

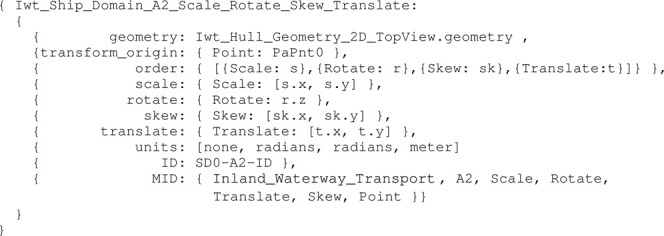

Several additional relations and constraints between aShip_Domain_A2 entity and other entities can be added. For example, the scale factors in Ship_Domain_A2 can be determined by the uncertainties of the proprioceptive sensor subsystem in Listing 14 (SD0). The models to apply at a certain point in time, given a specific situation, will be determined by the relations of the domains and corresponding constraints. Another example is to connect ship domain geometry with the de-acceleration motion model in Listing 12, or any other motion relation, as discussed in Section 3.3.3.2 for **SD1** and **SD2**.

#### 3.3.2 Map Domains

Currently, only two map domains are explicitly defined. These are:

• **MD0**: geometries, or collections of geometries, that form the static backbone of the map, i.e., the basemap. These are typically composed from external chart datasets, and generated during the map initialisation process, discussed in Section 3.2.1.
• **MD1**: all geometric entities that are dynamically added to the map at runtime, for example, the vessel and its ship domains belong to this level. These entities must contain relations with entities in MD0, and can moreover be divided into sub-domains, such as perception, navigation, control, other actors, map feature updates, and others. Generating this taxonomy is beyond the scope of this work.

#### 3.3.3 Experimental Design

Each experiment contains one or more elementary IWT situations (as seen in Table 1), consisting of semantic areas and actions (or manoeuvres). These situations are motivated by a series of real-world experiments, conducted over the last couple of years. It was found that many decisions were hard-coded in the software while they should have been available as externally configurable parameters. Consequently, the software proved to be hard to maintain and extend. While in these real-world scenarios, multiple, more complex situations can (co-)exist, this paper focusses on the simplest and most essential ones that can already improve interoperability and long-term developments significantly.

**TABLE 1:** Overview experiments: short description, main features, and reference to corresponding results.

	Description	Main feature(s)	Result	Example
Experiment 1	Introduction zeroth level domains for ship, **SD0**, and map, **MD0**	The uncertainty of the vessel position and its orientation determine the size of **SD0**; its shape is based on the vessel geometry	Section 4.1	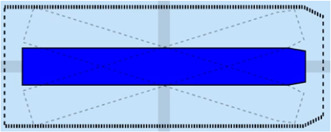
Experiment 2	Introduction of first, **SD1**, and second, **SD2**, level ship domains	The size of **SD1** corresponds to the minimally feasible breaking distance for the vessel, and its shape relates to the geometry of **SD0**. Similarly, **SD2** corresponds to the minimal speed decay distance	Section 4.2	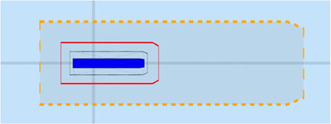
Experiment 3	Tolerances for **SD1** and **SD2** based on the hydrodynamic model	A predetermined tolerance factor scales the size of **SD1** and **SD2** based on whether or not the available hydrodynamic model of the vessel incorporates external forces (e.g., wind in this case)	Section 4.3	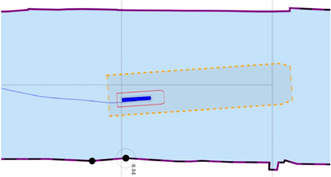
Experiment 4	The declaration of **SD2** as an anticipation zone	The shape of **SD2** will follow the navigational bounds of **MD0**, with a corresponding horizon configured by the user/operator	Section 4.4	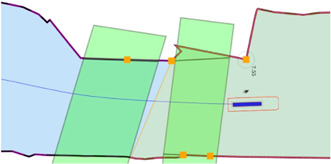
Experiment 5	Dynamic shortest-distance point features in **MD1**	Temporary features are added to **MD1** indicating the shortest linear distance between **SD0** and a point from **MD0** that has the navbounds meta tag	Section 4.5	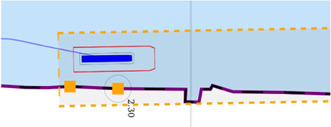
Experiment 6	The lane switch primitive added to **MD1**	A lane geometry is added to **MD1**, based on a set of relations between 1) the vessel entity, 2) its ship domains, 3) the COLREG meta model (avoiding collision in head-on situation), and 4) the map domains	Section 4.6	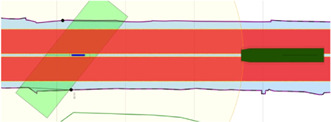
Experiment 7	Composability—integrated experiment combining several relations together	All the relations corresponding to previous experiments hold here as well, and are extended with a relation between the communication subsystem and **SD2**	Section 4.7	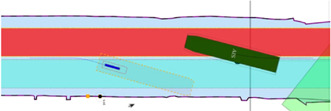

Furthermore, each experiment is discussed by means of providing 1) the context, including the motivation for the experiment, 2) a set of relations between the model-conform entities relevant within this context, and 3) a configuration, i.e., a description on how the models and relations are used in the situation(s).

##### 3.3.3.1 Experiment 1: The Axiomatic Body–Map Model Interaction

**Context:** A basic assumption for situational awareness in IWT, is a need for knowing a vessel’s pose within the physical world. Figure 2 visualises this basic interaction between the body and world models. In the physical world there is always an uncertainty associated with the pose of a vessel, as well as an accuracy of the local map. Furthermore note that pose uncertainty depends on accuracy and tolerances of proprioceptive sensors, possibly improved *via* exteroceptive sensors (see Figure 1C).

**Relations:** This experiment declares the **SD0** *via* a geometry–geometry relation. More precisely, the uncertainty of the vessel position and its orientation, provided by proprioceptive sensors, determine the size of **SD0** whereas its shape is based on the vessel geometry. The present IENCs have no explicit accuracy information available, hence **MD0** will be declared without uncertainty.

**Configuration:**

• Causal connection in visualisation of **SD0**: when the heading accuracy drives the width or length of the uncertainty zone, i.e., its size, an additional transparent vessel geometry rotated clockwise and counter-clockwise is plotted, in order to indicate that the vessel orientation causes the width of the ship domain. When the position accuracy determines the size of the domain, these additional rotated vessel geometries are not shown.
• The position for this ship domain in the map corresponds to Listing 17, with the shape according to Listing 18.
• To illustrate this causal link between the uncertainty source and the visualisation of **SD0**, the uncertainties in position (m) and heading (rad) i.e., (***ɛ***_***ν***_ = [*ϵ*_*x*_, *ϵ*_*y*_, *ϵ*_*θ*_]), change in the first 40 s of the simulation (see Section 4.1), as follows:

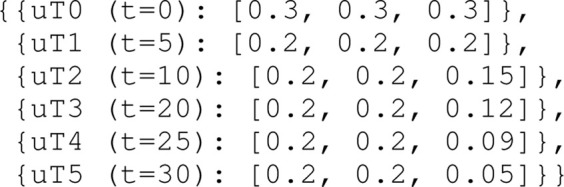


##### 3.3.3.2 Experiment 2: A Navigation Aid for (Remote) Operators and Control Systems

**Context:** The braking distance of a vessel, or its natural speed-decay distance, with corresponding polynomial coefficients in Listing 12, is crucial for a motion control system, or for a monitoring or controlling (onboard or remote) officer.

**Relations:** This experiment declares the **SD1** and **SD2** *via* a geometry–motion relation. More precisely, the size of **SD1** corresponds to the minimally feasible breaking distance for the vessel, whereas its shape relates to the geometry of **SD0**. Similarly, **SD2** corresponds to the minimal speed decay distance.

**Configuration:**

• Tolerance (in longitudinal and transversal direction), can be context-dependent on its own.
• Experimentally obtained third-order polynomials, with velocity as variable (using coefficients in Listing 12.
• Static reverse-motion tolerance, in addition to the pose uncertainty, in longitudinal and transversal direction. This small margin increases safety for motion-based decisions making.

##### 3.3.3.3 Experiment 3: Hydrodynamic Model Composition and Constraints

**Context:** Humans and robots should be able to unambiguously interpret the composition of the hydrodynamic model—and its constraints or assumptions—to operate and control the vessel, and to assign tolerances to specific control tasks accordingly, wherever needed. For example, especially for smaller vessels, such as the *Cogge*, it is generally important to account for wind whenever possible, and to *know* about whether or not wind is taken into account in the planned trajectories and short-term motion predictions of nearby vessels. If it is not, either by exclusion in the model, or by lack of an appropriate wind sensor, every application can decide for itself *how* to account for this shortcoming in the model.

**Relations:** This experiment adds tolerances to **SD1** and **SD2** *via* a geometry–motion relation. More precisely, when the hydrodynamic model of a vessel lacks the integration of wind forces, a predetermined tolerance factor scales the size of **SD1** and **SD2**. The semantic hydrodynamic model in Listing 9, has the external_forces property which includes the information of whether or not external forces, such as the wind forces, are included into its hydrodynamic model. This moreover imposes the constraint that the sensor subsystem has-a Iwt_Sensor_Wind entity.

**Configuration:**

• The tolerances can be dynamically activated, however, in this particular experiment, a human selects or deselect the checkbox, depending on whether external wind forces are included in the hydrodynamic model of the vessel or not (see Section 4.3).
• The determination of tolerances on the geometry of the vessel, more specifically, its surface above the waterline. The surface dimensions are factored with the static tolerance of the ship domains^9^.
• It should be noted that not only wind induces tolerance adjustments on the ship domains, however, for the purpose of *this* experiment, it is the only affecting parameter.

##### 3.3.3.4 Experiment 4: Cartoceptive Anticipation Domain

**Context:** Within the perception context, it can be useful to define explicit relations between a ship domain and other features in the semantic map. Such a ship domain could represent an area of interest for the vessel or operator. For example, this domain can be defined as an anticipation zone in which obstacles should be detected (within the navigation bounds of the map), to be used by the control system or operator.

**Relations:** This experiment declares **SD2** *via* a geometry–geometry relation. More precisely, the shape of **SD2** will follow the navigational bounds of **MD0**, and its size, or rather horizon, can be configured by the operator or the vessel.

**Configuration:**

• This experiment fetches all features that: 1) have the semantic tag (metadata) navbounds in the map, i.e., features related to the navigation bounds for the vessel, and 2) are within distance *U* from the vessel, with distance *U* in the direction Vessel_Principal_Axes_Frame.frame.u.
• This distance *U* is determined by the vessel, or operator, at runtime, and can be context-dependent on itself.
• A distance of 50 m in the sailing direction, and 20 m in the reverse direction is used.
• The resulting **SD2** domain is a Polygon composed from Points that belong to these navbounds features and fall within the range constraints of **SD2.**

##### 3.3.3.5 Experiment 5: Shortest Distance Between Uncertainty Zone and Navigation Boundary

**Context:** Knowledge of the distance between the vessel and an object, e.g., the shoreline, can augment situation awareness of the operator and/or the control systems.

**Relations:** This experiment adds temporary features to **MD1**—which indicate this shortest distance—*via* a main geometry–geometry relation. More precisely, a calculation determines the closest linear distance between **SD0** and a point from **MD0**, that is, a coordinate of a feature that has the semantic navbounds tag. As **MD0** features are currently not composed of symbolic, uniquely identifiable points, a temporary clone with the same coordinates is composed, and *does* get assigned a semantic tag. As such, this Point entity, i.e., aPntShortestDistNavbounds, can be used for online reasoning. For the experiments in this work, that means to determine whether aPntShortestDistNavbounds lies *within* **SD1** or **SD2**. This implies computing the Intersection between {**SD0**, **SD1**, **SD2**} and aPntShortestDistNavbounds.

**Configuration:**

**FIGURE 7:**
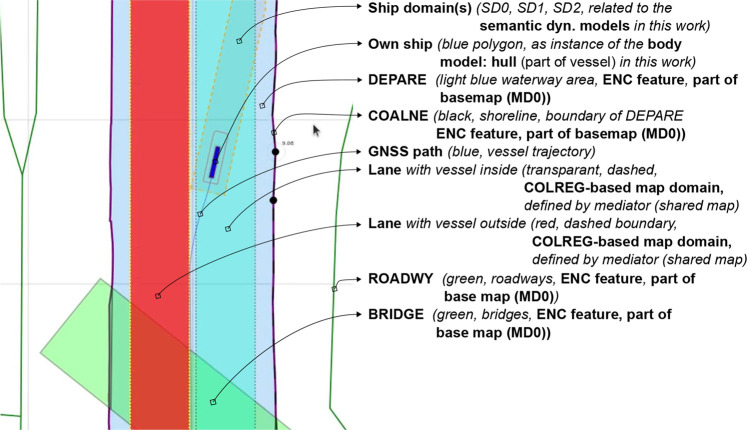
General legend for map features depicted throughout this paper.

•As discussed in Section 2.1, semantic metadata is only added to each map feature (e.g., LineString, Polygon, …) and not to each individual geometric map point. For example, the COALNE features (see also Figure 7) in the example map typically contain a LineString of 5–15 points, over a distance of 10–20 m.
•The associated navbounds are not yet treated as lines or polygons, but rather as the just-mentioned set of discrete points during the presently implemented shortest distance between **SD0**–**MD0** calculations.
•A temporary Semantic_ID is assigned to the Point that is part-of the Polygon geometry in Listing 8, and moreover holds the shortest distance relation with aPntShortestDistNavbounds in **MD0.**
•The following “intersection point representation” illustrate the result of the vessel–shoreline intersection calculation:
•{result: no intersection}: point = circle, black
•{result: intersect with **SD2**}: point = square, orange
•{result: intersect with **SD1**} : point = triangle, red
•A list of 6 *past* shortest points are kept as “recent history,” however, this value is dynamically configurable.
•Note that, presently, intersections with **SD0** trigger the same warning as an intersection with **SD1.** This need not be the case, however, their differentation falls out of the scope of the current experiment and work.

##### 3.3.3.6 Experiment 6: The Lane Shift Primitive for COLREGs

**Context:** COLREG Rule 14, head-on situation (a) states that: “when two power-driven vessels are meeting on reciprocal or nearly reciprocal courses so as to involve risk of collision, each shall alter her course to starboard so that each shall pass on the port side of the other.” To safely comply to this, and to other COLREG rules, especially in a highly automated environment, additional knowledge can significantly reduce confusion, and enhance safe, automated decision making to avoid collisions.

**Relations:** This experiment declares a lane shift primitive, based on a set of relations between 1) the vessel entity, 2) its ship domains, 3) the COLREG meta model, and 4) the map domains. The lane geometry, which is added to **MD1**, has a geometry–geometry relation with the navbounds-tagged entities in **MD0** (lane translation). Two additional geometry–geometry relations exists between **SD0** of the vessel entity, and the geometry of the lane, i.e., 1) to determine an intersection between these areas, and 2) to set the within constraint. A third, geometry–perception relation is present, where knowledge about the perception subsystem determines the geometry of **SD2**. Furthermore, the relation geometry–task is present, i.e. that the vessel’s task is to follow the lane in this particular situation, with corresponding implications for the controller.

**Configuration:**

• By default, when no objects are within the perception range, the lane in **MD1** is centred in the channel, with a width *w*_*lane*_ = *w*_*channel*_/*f*, where *f* is determined by the current situation, taking into account the relevant operational range (here 300 m). In this particular head-on case, *f* = 3.
• When a vessel’s **SD0** geometry is completely *within* the lane geometry, the lane has a “light green” colour. If not, the lane turns “red”. This visual confirmation can help (remote) operators to check whether they are in fact properly executing a task. The is-within relation offers similar knowledge for robots.
• Information about the exteroceptive sensor system, as modelled in Listing 14, helps determining an appropriate range for the circle geometry of **SD2**, in the geometry–perception context. The vessel velocity (see Listing 10) is also factored here. The perception range is divided into two sub-ranges, that is, a short range (0–50 m), and a long range (50–150 m). The LiDAR can detect movement and allows for a high-level classification of obstacles within the long range. At a relatively low forward speed ( <1m/s ), it can properly identify obstacles and corresponding geometries within the short-range. The visualised circle radius (**SD2**) is set to the short range, as this range is connected to the task execution (*discrete* control) of the vessel (in this case the lane shift execution). Data processing and context-reasoning within this horizon can still adjust the range of ship domain **SD2** at runtime, i.e., the range that triggers the lane switch.
• When another vessel’s **SD0** geometry is *within* the perception-related zone **SD2**, a lane switch is triggered, associated with an affine transformation of the centre lane(s), taking into account 1) COLREG rule 14, and 2) the width of the channel in **MD0.** This moreover triggers the controllers (of both vessels) to adjust their constraint values, in order to continue executing the “stay within the lane” task.
• It should be noted that several additional relations with considerable implications are *not* taken into account during in this experiment, such as:
• compliance to other COLREG rules, with respect to the width of the channel, or type of the vessel; and
• water depth information, as well as motion of the vessel, which can also have explicit (constraint) relations with the lane geometry.

##### 3.3.3.7 Experiment 7: Combination of Previous Experiments, With Additional Sensor-Subsystem Reasoning

**Context:** This experiment combines several of the above experiments. A vessel navigates a narrow channel, and passes two approaching vessels in opposite (head-on) directions. The first vessel *does* have AIS, and thus communicates its position to the *Cogge*, and the second vessel does not have AIS, meaning there is no way of knowing whether this second vessel is approaching without having a line of sight.

**Relations:** The abovementioned relations hold here as well. An additional relation between the Communication subsystem (AIS position of surrounding vessels), and the geometry of the cartoceptive **SD2** (own vessel), is added.

**Configuration:**

• By default, when no objects are within the short-range perception range (**SD2**), the lane in **MD1** is centred in the channel, similar to Section 3.3.3.6. The same *within* relation applies here as well.
• Perception horizon of 50–150 m is used to confirm movement of surrounding actors.
• When AIS position is communicated, the geometry–geometry relation between the cartoceptive ship domain **SD2** (similar to Section 3.3.3.4), and the AIS position and approaching vessel, triggers a lane switch is (AIS position is within the cartoceptive **SD2** geometry).
• When no AIS position is communicated, the geometry–perception related ship domain **SD2** (similar to Section 3.3.3.6), is used to trigger the lane switch.
• After passing a vessel, the geometry corresponding to the motion relation of **SD2** is used to trigger a “back-to-centre” lane switch, i.e., when the **SD0** domain geometry of the other vessel is-below the **SD2** motion-based geometry. This, together with the geometry–geometry relation between **MD0** and the vessel geometry in **MD1**, in combination with the motion of the vessel, determines whether a lane switch is appropriate.

### 3.4 Implementation

Implementation with respect to the content in this work includes (i) the models and their integration in an application context; (ii) solvers, such as: query solvers, solvers for kinematics and dynamics, optimisation engines, etc.; and (iii) situation monitoring and discrete control (performed by a mediator), e.g., higher-level services that: trigger the execution of the relevant solvers, provide constraints to the low-level (continuous) controllers, monitor a solver's quality and performance, among other things. Implementations related to the solvers are beyond the scope of this work. Actions and manoeuvres triggered by a mediator are to some extent described informally throughout Section 3.3.3, however, this work does not include their formal models and relations needed for higher-level reasoning. Hence, such actions and manoeuvres are still hard-coded in the software.

Consider Section 3.3.3.6 and Section 3.3.3.7, corresponding to the high-level structure in Figure 3, where a map is shared between two crossing vessels, and the mediator uses this map to determine the parameters to discretely control the head-on situation. Both vessels are able to interpret the models and relations that are used in the map composition. The shared map can be initialised by either one of the vessels, or by an external service. Furthermore, both vessels can have their own basemap(s), and/or their own map domains on top of the (shared) map. It is also important to note that the visualisation is always relative: this paper visualises the data from the perspective of the own-ship entity, however, local coordinate transformations, corresponding to other perspectives, result in different visualisations of the exact same data structures. When Vessel A initialises the shared map, that is, offering the model-conform data structures in a particular situation, and Vessel B is familiar with these models, both entities can agree on using the map for mediating this situation. For this lane shift primitive, the model-conform data structures include: 1) the static navigation bounds (from the navigational charts), 2) **SD0** of both vessels, 3) and the lane geometries and associated relations, e.g., with the motion constraints of both vessels. When linked with the perception subsystem, this list can be extended with relations to dynamic obstacles and their geometries. The dynamically composed lane geometries and situation-specific policies^10^ can in turn be used as control constraints for the respective vessels to perform COLREG-compliant, hence, collision-free manoeuvres. The internal processing, integration in the controller, and general implementation, are completely up to the user, as long as all constraints are met. Such constraints can be imposed by a mediator, or by any of the relations, both internally and externally, relevant in a certain situation. The lane geometry and corresponding lane-shift procedure could be updated dynamically, for example, when the perception subsystem of Vessel A detects an obstacle^11^. Even if only Vessel A is able to detect such obstacles, these kind of events tend to have implications for both vessels, e.g., by updating the lane geometries in the shared map, at runtime. As long as the world models and relations are known by both actors, such discrete control decisions can be understood/explained, and justified.

Models are implemented using flatbuffer schemas, or human-readable JSON-LD schemas (or both), depending on the application layer. JSON-LD offers a modelling standard for graph-structured relations, as it introduces additional property types such as @id to assign a model ID, @context for meta model connections, and @type for meta model conforms-to relations. In addition to flatbuffers and JSON-LD, this work adopts the SVG-standard to compose the semantic map^12^. This standard already offers a set of standardised primitives to define and compose geometric features. SVG elements can have a symbolic identifier (id), and (meta) model tags can be added to their class-list. Additionally, this approach offers a straightforward query interface for free (next to querying spatial features from a database, for example), directly on the shared map itself, using Javascript’s built-in querySelector method in a situation-related scope in the Document Object Model (DOM) tree. The SVG can moreover be directly rendered, for instance, in a browser. For realtime exchange of model-conform data structures, flatbuffers are preferred considering their performance benefits, as they allow for compressed payloads and zero-copy deserialisation of the data. Moreover, these flatbuffers are defined using an interface description language that is compatible to the protocol buffer format (.proto). Naturally, many other data formats could be used as well.

#### 3.4.1 Assumptions

All experiments are conducted using systems that are capable of interpreting a minimal number of data structures conform to the models discussed in this work. Currently, no validation service is implemented to check whether or not a system is actually compliant. On the application layer, a simple boolean (always true in our case) determines whether an actor can interpret the data associated with a certain model. This implies that non-compliant actors with respect to the semantic map are not allowed to dynamically add/update features to/on a *shared* map. Similar to a shared map, a realtime peer-to-peer stream of hydrodynamical data, perception data, or any other relevant piece of information for that matter, can be set up between actors, as long as appropriate (meta) model information is exchanged at the initial handshake to guarantee correct interpretation (again, always assumed true here).

Regarding shared updates, a boolean property is added to the meta data of a model-conform data structure. This allows actors that produce such data to indicate whether or not the data may be updated by other compliant actors in the same environment. A relevant example could be the geometry of the bounding box of a detected obstacle in a particular environment. Distributed data structure updates can be rather complex, and will require additional policies, models, and some kind of validation or voting system. This, however, is beyond the scope of this work, that aims to focus on the building blocks for such future developments. The same limitation holds for validation of uncertainties, i.e., **SD0**, of various objects, including vessels. This is closely related to the perception-subsystem of the vessel. Consequently, additional models within this very context are necessary.

## 4 Results

The results of the above-designed experiments can be found in the supplementary material videos in Section 5.1. This section briefly shows a few core snapshots of each video to illustrate the results. An overview was provided in Table 1. These snapshots include (some of) the following features shown in Figure 7. In Section 4.1, Section 4.2, Section 4.3, Section 4.4, the main vessel entity is considered the mediator, as it determines its related ship domains according to information provided by its body-model subsystems. In Section 4.5, an additional higher-level mediator is introduced, i.e., an operator controlling the vessel in open loop, where relations between the body model and map are used, together with internal world model-based information, to determine appropriate actuation inputs. Lastly, in Section 4.6, Section 4.7, the semantic map, which is shared between various actors, is used by a situation monitoring service to mediate the situation. The additional lanes are added to the map at runtime, and hence, the map influences continuous control tasks and constraints for each actor.

### 4.1 Experiment 1

**FIGURE 8:**
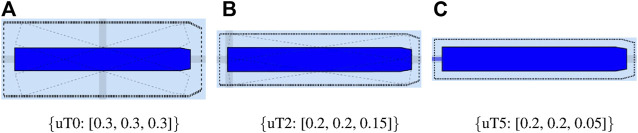
Dynamic behaviour of the axiomatic **SD0** for the different time steps and associated uncertainties.

Figure 8 shows three snapshots of the Supplementary Video S1 which demonstrates the causal relation between the vessel’s pose uncertainty and its **SD0**.

### 4.2 Experiment 2

**FIGURE 9:**
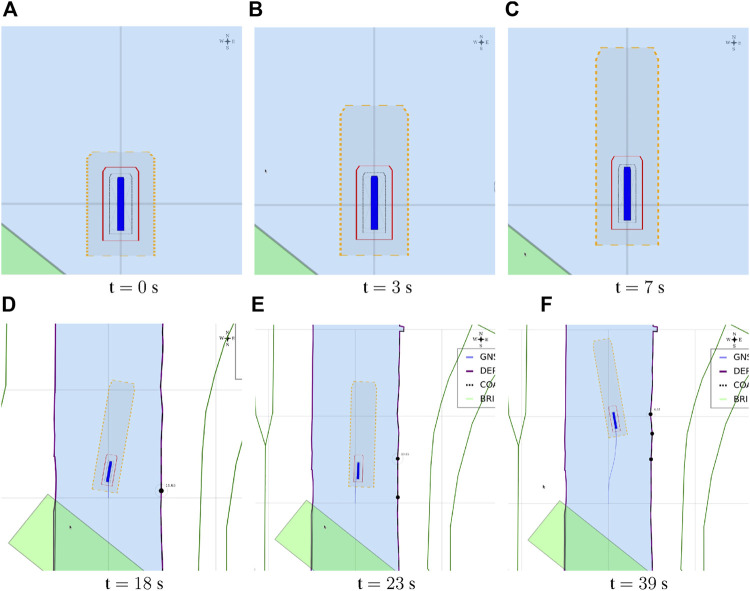
Dynamic behaviour of **SD1** and **SD2** based on the hydrodynamic motion model of the vessel.

Figure 9 shows several snapshots of the Supplementary Video S2 which introduces **SD1** and **SD2** based on the hydrodynamic motion model of the vessel.

In this experiment, and in all subsequent experiments, the constraint in Listing 11 is applied to the hydrodynamic model. When the forward velocity of the vessel is below a certain threshold (0.5 m/*s*), the damping is linear, whereas above, it is linear + non-linear. This is especially important when the vessel has to perform specific, complex, tasks at low speeds (docking, mooring). When the 3DOF model of Listing 9 is used inside the control loop (e.g., for model predictive control), at low speeds, the model is very sensitive to the non-linear damping matrix, and will not mimic the physical behaviour of the vessel. Similarly, when encountering one or multiple vessels in a canal, or at a terminal, a mediator needs to know whether the models used to execute the respective control tasks of systems involved are adjustable to the context and motion of the vessel. Not having this information could lead to unexpected behaviour, such as unfeasible control objectives resulting in collisions.

### 4.3 Experiment 3

**FIGURE 10:**
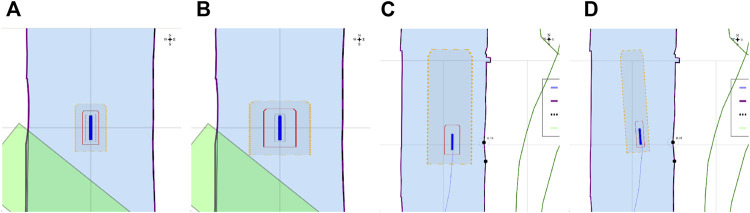
Dynamically allocated tolerances to **SD1** and **SD2** *via* a main geometry–motion relation (with hydrodynamical model of the vessel), both for a static vessel **(A,B)** and a moving vessel **(C,D)**.

Figure 10 shows four snapshots of the Supplementary Video S3 which illustrates the effect of the incorporation of the external wind forces on **SD1** and **SD2**.

For the simulator, by default, wind is modelled. The video shows how gusts of wind (of 10 m/*s*, or 4–5 Beaufort), can have a significant impact on the behaviour of the vessel. For example, the vessel drifts several meters in the x-direction without any thrust forces applied in this direction.

### 4.4 Experiment 4

**FIGURE 11:**
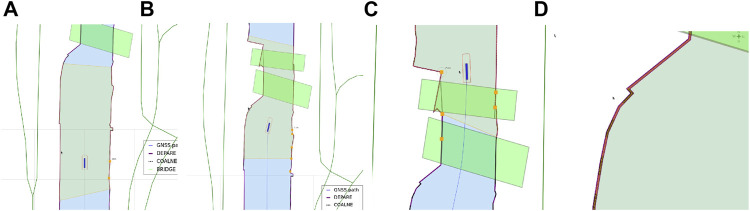
Anticipation domain declaration, i.e., the zone in which obstacles should be detected (within the navigation bounds of the map), of **SD2** *via* a main cartoceptive geometry–geometry relation.

Figure 11 shows four snapshots of the Supplementary Video S4 which illustrates the anticipation domain of **SD2** as a cartoceptive relation with the navbounds features in **MD0**. These domains can help a robot anticipate by triggering discrete control tasks based on geometry relations such as within or intersect. For example, when no perception sensors are available, and another vessel’s AIS position claims to be within this range, it can trigger a lane shift, as demonstrated in Section 4.7. Another use case for this domain is to detect, or link, known shoreline landmarks, e.g., Points integrated in the map, in the perception sensor data (geometry–perception), or vice versa.

### 4.5 Experiment 5

**FIGURE 12:**
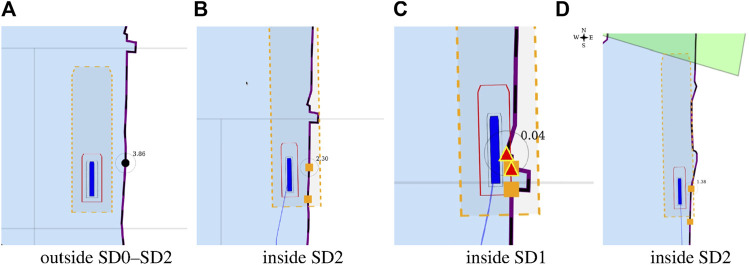
Dynamic shortest distance between vessel and shoreline additions to **MD0** *via* a main geometry–geometry relation.

Figure 12 shows four snapshots of the Supplementary Video S5 which illustrates the shortest distance between the Point entities in vessel’s **SD0** and the local coordinates of the navbounds features in **MD0**. By means of dynamically allocating a semantic tag to each closest Point entity, it allows vessel (sub)systems, as well as other actors within the operating range, to unambiguously identify this point, as part of a feature. Furthermore, it can be seen that whenever the vessel’s danger zone **SD1**, related to the minimum aided deceleration distance (see Listing 12), intersects with the shoreline in **MD0**, the vessel almost hits the shoreline. The added tolerances on this zone keep the vessel from colliding with the shoreline, although this particular scenario should, obviously, be avoided when executing the move-along-channel task.

### 4.6 Experiment 6

**FIGURE 13:**
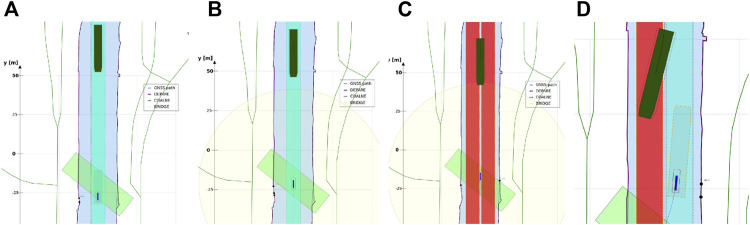
Lane shift primitive, triggered by geometry–perception relation (exteroceptive sensor subsystem), i.e., an approaching vessel detected by exteroceptive sensor(s), within predefined range. Lanes with vessel *inside* is shown in transparent green; lanes with vessels *outside* are shown in red.

Figure 13 shows four snapshots of the Supplementary Video S6 which illustrates the lane shift primitive. This lane shift primitive, integrated in **MD1**, provides semantic information in the form of additional geometric entities and constraint relations for the vessel to simply follow the COLREG rule in a highly automated environment.

### 4.7 Experiment 7

**FIGURE 14:**
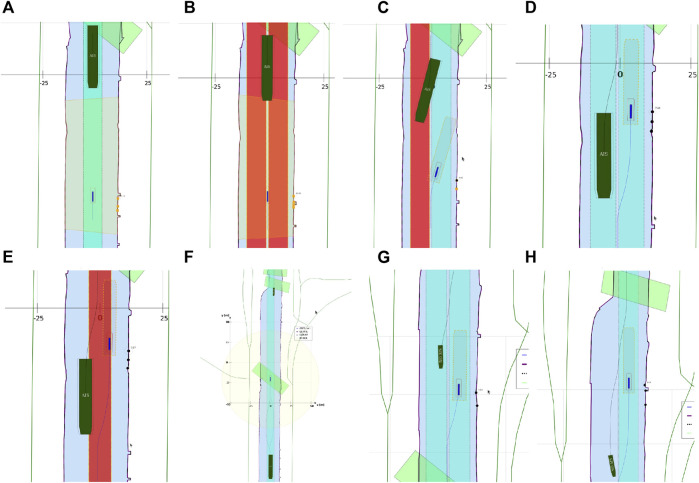
Combination of situations and relations of previous experiments, with additional sensor subsystem relations.

Figure 14 lists eight snapshots of the Supplementary Video S7 which illustrates a combination of situations and relations discussed in the previous experiments.

After passing the first vessel, the geometry corresponding to the motion relation of **SD2** triggers a “back-to-centre” lane switch. After passing the second vessel, the mediator uses the geometry–geometry relation between **MD0** and **MD1**, in combination with the current position of the vessel in the map, to overrule the “back-to-centre” lane switch. Additional knowledge about the lane switch, i.e., switching lanes takes about 25 m at this speed, determines that a lane switch *before* the channel narrowing is not very efficient, or safe. Note that in this case, it is still feasible. Therefore, in the second case, the vessel stays in the switched side-lane for passing the bridge, based on the cartoceptive information provided by the semantic map.

The shared semantic map is used by the mediator, who acknowledges the fact that both actors are able to interpret the map-based constraints unambiguously in order to proceed. Once the agreement is reached, the information provided by the map, given there is sufficient, model-compliant input from each actor, could be used in the discrete controllers of the respective vessels. In this case, the dynamic lane geometry is used as a set of controller constraints. This includes geometric constraints as well as tolerances, desired navigation direction for each lane, speed limits, among other relevant (shared) data.

## 5 Discussion

This paper presents a set of semantic world models within the context of Inland Waterway Transport. The models are a first step towards the formalisation of various situations that can occur on a waterway, focusing on the simplest and most essential ones. Each situation consists of 1) a set of “semantic areas” (represented by geometrical polygons on a basemap, with symbolic labels), and 2) a set of “actions,” for motion, perception, and information processing. The models are inter-connected, by so-called “higher-order relations,” to support the creation of a “context.” The paper focuses on making the models in such a way that 1) their granularity is small enough to compose models together in higher-order models without loss of interpretability, 2) they form the basis for inter-vessel messaging, and 3) (hence) result in explainable engineering systems and higher levels of shared automation.

The elementary situations discussed in the experiments were motivated by a range of real-world experiments, performed over the past couple of years. Developing the software for these real-world experiments turned out to be hard to maintain and extend, because so many decisions were hard-coded in the software while they should have been available as externally configurable parameters.

In the context of this paper, the formal extensions have not yet been tested on the real vessels, because their on-board control software is not (yet) capable of exploiting them. So, to illustrate the added value of the formalisations, a simulation of the vessels was used, with real-world navigation maps as basemaps, and simplified motion models for the vessels. In such simulation environments, it is rather straightforward to make all entities in a certain local environment compliant to a set of world models. In reality, this will rarely be the case, hence, research on how to be robust against “unknown” entities and features is required. The envisaged approach to reach such robustness is to extend the number of situations, linking them together with higher-order relations that encode how a more complex situation conforms to a composition of simpler situations together with a particular set of disturbances.

As a next step, new real-world experiments will be conducted in a *controlled* environment, that is, an environment in which only model-conform entities operate, such as vessels from our research fleet. Since our experimental facilities lie in an area where all of the simulated situations in this paper exist in reality, similar real-world experiments can be performed. To this end, a perception-based control strategy without any semantic reasoning will serve as a benchmark. Then, additional experiments with actors that have access to a cartoceptive subsystem, i.e. a (shared) semantic map, and corresponding world models, will test against that benchmark. These experiments will be performed with both remote controlled vessels, and with autonomous vessels. Regarding the former, it is expected that visual feedback of, for instance, a lane shift primitive, will help the remote operator to safely (smaller error margins) and efficiently (smaller manoeuvre time) navigate the vessel. With respect to the latter, it is expected that such shared, discrete control functionality (provided by a mediator) will reduce overall complexity of the highly automated environment, and as such improve safety, explainability and performance. Especially in close encounter manoeuvring, higher-level control decisions influenced by the shared map are expected to allow a vessel to quicker anticipate, and thus better avoid potential collisions.

### 5.1 Conclusion

The simulation experiments in this paper corroborate the research hypothesis of the authors that the investment in formalising IWT situations adds value to the automation of (semi-)autonomous shipping. And that starting with the formalisation of the “traffic rules” in the waterways is a very appropriate starting point: the existing commercial navigation maps provide the basemap information of the geometrical layout of the waterways, so that we can add “semantic traffic areas” on top of the map, as well as the situations that are relevant in such areas.

A collaborative effort from research institutions, standardisation committees, and the IWT industry is a necessary next step to develop a workable set of models and relations that can be tested and integrated in daily operations. The existence of such internal world models of the robot, and external world models of a local environment, can significantly enhance safe, cost-effective (shared) automation procedures. It provides a necessary complementary extension to the existing legacy models and standards, such as AIS or NMEA. As such, it can accommodate higher levels of automation, as well as better task anticipation by humans and robots. Explainable operational (meta) data will help a mediator to reason about particular situations, and take over control whenever necessary. Furthermore, formal models can be a crucial part in regulatory frameworks for IWT. The allowed level of automation for a robot can depend on its capability to comply to a formal set of standardised models, and thus to be able to justify its automated decision making to a reasonable extent. As such, improving, and extending world models in close collaboration with policy makers, waterway administrators, and IWT companies, is a challenging goal for the near future.

## Data Availability

The original contributions presented in the study are included in the article/Supplementary Material, further inquiries can be directed to the corresponding author.

## Footnotes

1 It is important to note here that a failure to take early action—i.e., without malintent—is merely a consequence of the ability to take early action, hence, it fully relies on SA.

2 Although the COLREGs, i.e., regulations for decision making, have been designed to determine vessel manoeuvres without requiring any communication between the vessels, it does demand the use of all available means to obtain optimum situational awareness (COLREGs rule 5) (Tsimplis and Papadas, 2019).

3 A higher-order relation is a relation about a relation, that is, a relation that has other relations as its entities, or “arguments.”

4 The words symbolic and descriptive, both referring to the model representation, are used interchangeably throughout this work.

5 An optional, third, meta meta model ID (MMID) can be used: a set of unique identifiers that each point to a meta meta model of this model, needed to transform the model to another formal representation, while keeping the meaning of the model unchanged. However, this is *not* included in this work.

6 the GeoJSON format contains the following geometric objects: “Point”, “MultiPoint”, “LineString”, “MultiLineString”, “Polygon”, “MultiPolygon”, and “GeometryCollection”.

7 In this work, a mereo-logical representation refers to the simplest interpretation (or also the “highest” level of abstraction). A mereo-logical model just represents the parts that make up the world, without any additional structure or behaviour. Hence, the meta model of a mereo-logical model is trivial: a list of acceptable keywords.

8 Various standardised data formats can be used to formally describe elements, e.g., the JSON or XML schemas. For this text, some minor adjustments to the JSON syntax are applied to increase readability.

9 The experiments in this work use scaling factors inspired by wind-modelling in Fossen (1994), i.e., corresponding to the vessel's surface geometries above the waterline, i.e., 1.12 (1+*h*_*v*,*aw*_ × *l*_*v*_) in the longitudinal direction, and 1.96 (1+*h*_*v*,*aw*_ × *w*_*v*_) in the and transversal direction.

10 Situation-specific policies include issuing a warning whenever a vessel’s **SD0** threatens to exceed the lane boundaries (+ tolerance). If no correction occurs, and the manoeuvre is still ongoing, termination of the manoeuvre is required, and some “safe fallback mode” could be enabled. As seen in Result 4.6, part of this policy is visualised on the map by changing the colour of the lanes.

11 Object detection *via* the perception sensor subsystem is not simulated, hence, dynamic updates of the lane geometry are not part of the experiments discussed in this work.

12 Composing an SVG map from various lower-level models *can* involve model-to-model transformations, e.g., between the JSON-LD standard and the SVG standard.

## Supplementary Material

Supplementary Video S0 Research Vessel Cogge in operation.

Supplementary Video S1 Video—Introduction zeroth level ship and map domain.

Supplementary Video S2 Video—Motion based **SD1** and **SD2.**

Supplementary Video S3 Video—Tolerances for **SD1** and **SD2** based on the hydrodynamic model with or without wind.

Supplementary Video S4 Video—Cartoceptive geometry relation for **SD2** based on navbounds map features in **MD0.**

Supplementary Video S5 Video—Shortest distance, based on the cartoceptive geometry relation with **SD0**, i.e., its Intersection with navbounds map features in **MD0.**

Supplementary Video S6 Video—Lane shift primitive, in compliance with COLREG head-on vessel operations.

Supplementary Video S7 Video—Combination of previous experiments, with additional sensor subsystem relations.

## Abbreviations

IW, Inland Waterway(s); IWT, Inland Waterway Transport; SD, Ship Domain; MD, Map Domain; (I)ENC, (Inland) Electronic Navigational Charts.

## Appendices

### A Hydrodynamic motion model Cogge

#### A.1 Full components form

$$\begin{array}{c} \left(\underset{M_{RB}}{\underset{︸}{\left[\begin{array}{ccc} m & 0 & 0\\ 0 & m & mx_{g}\\ 0 & mx_{g} & I_{z} \end{array}\right]}}+\underset{M_{A}}{\underset{︸}{\left[\begin{array}{ccc} -X_{\dot{u}} & 0 & 0\\ 0 & -Y_{\dot{v}} & -Y_{\dot{r}}\\ 0 & -N_{\dot{v}} & -N_{\dot{r}} \end{array}\right]}}\right)\left[\begin{array}{c} \dot{u}\\ \dot{v}\\ \dot{r} \end{array}\right]\\ +\left(\underset{C_{RB}}{\underset{︸}{\left[\begin{array}{ccc} 0 & 0 & -m\left(x_{g}r+v\right)\\ 0 & 0 & mu\\ m\left(x_{g}r+v\right) & -mu & 0 \end{array}\right]}}+\underset{C_{A}}{\underset{︸}{\left[\begin{array}{ccc} 0 & 0 & Y_{\dot{v}}v+\frac{1}{2}\left(Y_{\dot{r}}+N_{\dot{v}}\right)r\\ 0 & 0 & -X_{\dot{u}}u\\ -Y_{\dot{v}}v-\frac{1}{2}\left(Y_{\dot{r}}+N_{\dot{v}}\right)r & X_{\dot{u}}u & 0 \end{array}\right]}}\right)\left[\begin{array}{c} u\\ v\\ r \end{array}\right]\\ +\left(\underset{D_{N}}{\underset{︸}{\left[\begin{array}{ccc} -X_{|u|u}|u| & 0 & 0\\ 0 & -Y_{|v|v}|v| & -Y_{|v|r}|v|\\ 0 & -N_{|v|v}|v| & -N_{|v|r}|v| \end{array}\right]}}+\underset{D_{L}}{\underset{︸}{\left[\begin{array}{ccc} -X_{u} & 0 & 0\\ 0 & -Y_{v} & -Y_{r}\\ 0 & -N_{v} & -Nr \end{array}\right]}}\right)\left[\begin{array}{c} u\\ v\\ r \end{array}\right]=\left[\begin{array}{c} \tau_{X}^{c}\\ \tau_{Y}^{c}\\ \tau_{N}^{c} \end{array}\right]+\left[\begin{array}{c} \tau_{X}^{w}\\ \tau_{Y}^{w}\\ \tau_{N}^{w} \end{array}\right] \end{array}$$
(10)

With:

$$\tau_{control}=\left[\begin{array}{c} \tau_{X}^{c}\\ \tau_{Y}^{c}\\ \tau_{N}^{c} \end{array}\right]=\left[\begin{array}{c} T_{j}^{x}\left(n_{j},\alpha_{j}\right)+T_{c}^{x}\left(n_{c},\alpha_{c}\right)\\ T_{j}^{y}\left(n_{j},\alpha_{j}\right)+T_{c}^{y}\left(n_{c},\alpha_{c}\right)\\ L_{j}^{x}\times T_{j}^{y}\left(n_{j},\alpha_{j}\right)+L_{c}^{x}\times T_{c}^{y}\left(n_{c},\alpha_{c}\right) \end{array}\right],\tau_{wind}=\left[\begin{array}{c} \tau_{X}^{w}\\ \tau_{Y}^{w}\\ \tau_{N}^{w} \end{array}\right]=\left[\begin{array}{c} \ldots \\ \ldots \\ \ldots \end{array}\right].$$
(11)

#### A.2 Identified hydrodynamic coefficients

As mentioned in Section 3.1.3, the hydrodynamic model of *the Cogge* has threshold speed values (see Listing 11), which trigger the configuration of the damping matrices. For the higher velocity regime of *the Cogge*, meaning, $u>0.5\frac{\text{m}}{\text{s}}$ , and $v>0.15\frac{\text{m}}{\text{s}}$ , the value of the coefficients for **M**_*RB*_, **C**_*RB*_, **M**_*A*_, **C**_*A*_, **D**_*N*_, and **D**_*L*_ can be found in the Listing 9. The diagonal terms (except *N*_|*v*|*r*_|*v*|) of these matrices are based on the $M\left(\theta_{u}^{e}\right)$ , $M\left(\theta_{v}^{e}\right)$ , and $M\left(\theta_{r}^{f}\right)$ model structures, identified and discussed in Peeters et al., (2020c). The remaining off-diagonal terms were manually tuned with reference to a port and starboard side turning circle.

For the lower speed regime, **D**(***ν***) switches from **D**_**N**_ + **D**_**L**_ to **D**_**L**_. Accordingly, all the terms of **D**_**N**_ become zero. The terms of **D**_**L**_ change to *X*_*u*_ = 39.3, *Y*_*v*_ = 226.31, based on $M\left(\theta_{u}^{f}\right)$ , $M\left(\theta_{v}^{f}\right)$ from Peeters et al., (2020c), note that the yaw motion already had a linear damping with respect to the yaw-rate.

As mentioned in Section 2.4, bollard pull thrust models suffice in the context of this work. Therefore, two look-up-table actuation models were implemented based on the bollard pull data discussed in Peeters et al., (2020a). Note that for the stern thruster, the data were cleaned accordingly: constant propeller rate for each angular data set, and the assumption that the thrust force at zero degrees control angle is aligned with this angle.

Hydrodynamic coefficients used in this work are shown in Listing 19.

**Listing 19:** Hydrodynamical coefficients

## References

[B1] Argüelles R. P. García Maza J. A. Martín F. M. Bartolomé M. (2021). Ship-to-ship Dialogues and Agreements for Collision Risk Reduction. J. Navigation, 1–18.

[B2] AUTOBarge (2021). The Autobarge Project. Available at: https://etn-autobarge.eu/project/ .

[B3] AVATAR (2021). The Avatar Project by the North Sea Region Programme 2014–2020. Available at: https://northsearegion.eu/avatar/. AVATAR is a project co-funded .

[B46] Bakillah M. Liang S. H. L. Zipf A. Mostafavi M. A. (2013). A Dynamic and Context-Aware Semantic Mediation Service for Discovering and Fusion of Heterogeneous Sensor Data. J. Spat. Informat. Sci. 6, 155–185. 10.5311/JOSIS.2013.6.104

[B4] Blanke M. (1981). Ship Propulsion Losses Related to Automatic Steering and Prime Mover Control. Ph.D. thesis. Oxford: Technical University of Denmark. 87-87950-14-6.

[B5] Bruyninckx H. (2021). Design of Complicated Systems (Online Book). Work in Progress. Available at: https://robmosys.pages.gitlab.kuleuven.be/ .

[B6] Essen H. V. Schroten A. Otten M. Sutter D. Schreyer C. Zandonella R. (2016). Developping a Cost Calculation Model for Inland Navigation. Amsterdam: Research in Transportation Business & Management, 64–74.

[B7] European Commission (2019). Mobility and Transport: Promotion of Inland Waterways. Tech. rep.

[B8] Fedyaevsky K. K. Sobolev G. V. (1964). Control and Stability in Ship Design. Leningrad: State Union Shipbuilding Industry Publishing House.

[B9] Fossen T. I. Fjellstad O.-E. (1995). Nonlinear Modelling of marine Vehicles in 6 Degrees of freedom. Math. Model. Syst. 10.1080/13873959508837004

[B10] Fossen T. I. (1994). Guidance and Control of Ocean Vehicles. John Wiley & Sons.

[B11] Fossen T. I. (2011). Handbook of Marine Craft Hydrodynamics and Motion Control. Chichester, UK: John Wiley & Sons, Ltd.

[B12] Fossen T. I. (1991). Nonlinear Modeling and Control of Underwater Vehicles. phdthesis: Norwegian Institute of Technology.

[B13] Fujii Y. Tanaka K. (1971). Traffic Capacity. J. Navigation 24, 543–552. 10.1017/s0373463300022384

[B14] Hansen M. G. Jensen T. K. Lehn-Schiøler T. Melchild K. Rasmussen F. M. Ennemark F. (2013). Empirical Ship Domain Based on Ais Data. J. Navigation 66, 931–940. 10.1017/s0373463313000489

[B15] Huang Y. Chen L. Chen P. Negenborn R. R. van Gelder P. H. A. J. M. (2020). Ship Collision Avoidance Methods: State-Of-The-Art. Saf. Sci. 121, 451–473. 10.1016/j.ssci.2019.09.018

[B16] IMO (2001). Resolution A.918(22). Tech. rep. in Standard Marine Communication Phrases (International Maritime Organization).

[B17] Isherwood R. M. (1972). Wind Resistance of Merchant Ships. RINA Transcripts 115, 327–338.

[B18] Iw-Net (2021). The Iw-Net Project from the European Union’s Horizon 2020 Research and Innovation Programme under grant Agreement No 861377. Available at: https://www.isl.org/en/projects/iw-net. This project has received funding .

[B19] Kallas S. (2011). Transport 2050: Comission Outlines Ambitious Plan to Increase Mobility and Reduce Emmisions. Tech. Rep. March.

[B20] Kotzé M. Junaid A. B. Afzal M. R. Peeters G. Slaets P. (2019). Use of Uncertainty Zones for Vessel Operation in Inland Waterways. J. Phys. Conf. Ser. (Bristol: IOP Publishing), Vol. 1357, 012031. 10.1088/1742-6596/1357/1/012031

[B21] Landsiedel C. Rieser V. Walter M. Wollherr D. (2017). A Review of Spatial Reasoning and Interaction for Real-World Robotics. Adv. Robotics 31, 222–242. 10.1080/01691864.2016.1277554

[B22] Lang D. Friedmann S. Haselich M. Paulus D. (2014). Definition of Semantic Maps for Outdoor Robotic Tasks. 2014 IEEE International Conference on Robotics and Biomimetics (ROBIO 2014). IEEE, 2547–2552. 10.1109/robio.2014.7090724

[B23] Lewis E. V. (1989). Principles of Naval Architecture. in Resistance, Propulsion and Vibration. 2nd rev. ed. edn (Jersey City, NJ: Society of Naval Architects and Marine Engineers), Vol. 2.

[B24] Liu J. Zhou F. Li Z. Wang M. Liu R. W. (2016). Dynamic Ship Domain Models for Capacity Analysis of Restricted Water Channels. J. Navigation 69, 481–503. 10.1017/s0373463315000764

[B25] Nüchter A. Hertzberg J. (2008). Towards Semantic Maps for mobile Robots. Robotics Autonomous Syst. 56, 915–926.

[B26] Peeters G. Afzal M. R. Vanierschot M. Boonen R. Slaets P. (2020a). Model Structures and Identification for Fully Embedded Thrusters: 360-Degrees-Steerable Steering-Grid and Four-Channel Thrusters. Jmse 8, 220. 10.3390/jmse8030220

[B27] Peeters G. Kotzé M. Afzal M. R. Catoor T. Van Baelen S. Geenen P. (2020b). An Unmanned Inland Cargo Vessel: Design, Build, and Experiments. Ocean Eng. 201, 107056. 10.1016/j.oceaneng.2020.107056

[B28] Peeters G. (2021). Towards Unmanned Inland Shipping. phdthesis, KU Leuven. Available at: https://lirias.kuleuven.be/3391744?limo=0 .

[B29] Peeters G. Van Baelen S. Yayla G. Catoor T. Afzal M. R. Christofakis C. (2020c). Decoupled Hydrodynamic Models and Their Outdoor Identification for an Unmanned Inland Cargo Vessel with Embedded Fully Rotatable Thrusters. Jmse 8, 889. 10.3390/jmse8110889

[B30] Peeters G. Yayla G. Catoor T. Van Baelen S. Afzal M. R. Christofakis C. (2020d). An Inland Shore Control centre for Monitoring or Controlling Unmanned Inland Cargo Vessels. J. Mar. Sci. Eng. 8. 10.3390/jmse8100758

[B31] SINTEF (2020). The hull-to-hull (H2h) Project from the European GNSS Agency under the European Union’s Horizon 2020 Research and Innovation Programme grant Agreement No 775998. Available at: https://www.sintef.no/projectweb/hull-to-hull/. This project has received funding .

[B32] SNAME (1950). “Nomenclature for Treating the Motion of a Submerged Body through a Fluid,”. Technical and Research Bulletin (Jersey City, NJ: The Society of Naval Architects and Marine Engineers), 1–5. Tech. Rep.

[B33] Sotiralis P. Ventikos N. P. Hamann R. Golyshev P. Teixeira A. P. (2016). Incorporation of Human Factors into Ship Collision Risk Models Focusing on Human Centred Design Aspects. Reliability Eng. Syst. Saf. 156, 210–227. 10.1016/j.ress.2016.08.007

[B34] Sys C. Vanelslander T. (2011). Future Challenges for Inland Navigation. No. 978 90 5487 845 4 in (UPA).

[B35] Szlapczynski R. Szlapczynska J. (2017). Review of Ship Safety Domains: Models and Applications. Ocean Eng. 145, 277–289. 10.1016/j.oceaneng.2017.09.020

[B36] Tavasszy L. A. Behdani B. Konings R. (2015). Intermodality and Synchromodality. Available at: https://papers.ssrn.com/sol3/papers.cfm?abstract_id=2592888 .

[B37] The European Commission (2019). Watertruck+. Available at: http://www.watertruckplus.eu/ .

[B38] Tsimplis M. Papadas S. (2019). Information Technology in Navigation: Problems in Legal Implementation and Liability. J. Navigation 72, 833–849. 10.1017/s0373463318001030

[B39] Tsou M.-C. (2016). Multi-target Collision Avoidance Route Planning under an Ecdis Framework. Ocean Eng. 121, 268–278. 10.1016/j.oceaneng.2016.05.040

[B40] Ung S.-T. (2019). Evaluation of Human Error Contribution to Oil Tanker Collision Using Fault Tree Analysis and Modified Fuzzy Bayesian Network Based Cream. Ocean Eng. 179, 159–172. 10.1016/j.oceaneng.2019.03.031

[B41] van Essen H. (2018). Sustainable Transport Infrastructure Charging and Internalisation of Transport Externalities. Tech. Rep. December.

[B42] Verberght E. (2019). INN-IN: Innovative Inland Navigation. Tech. Rep. University of Antwerp, Department of Transport and Regional Economics.

[B43] Wang Y. Chin H.-C. (2016). An Empirically-Calibrated Ship Domain as a Safety Criterion for Navigation in Confined Waters. J. Navigation 69, 257–276. 10.1017/s0373463315000533

[B44] Weng J. Yang D. Chai T. Fu S. (2019). Investigation of Occurrence Likelihood of Human Errors in Shipping Operations. Ocean Eng. 182, 28–37. 10.1016/j.oceaneng.2019.04.083

[B45] Yildirim U. Basar E. Ugurlu O. (2019). Assessment of Collisions and Grounding Accidents with Human Factors Analysis and Classification System (Hfacs) and Statistical Methods. Saf. Sci. 119, 412–425.

